# Detection of Diffusion Heterogeneity in Single Particle Tracking Trajectories Using a Hidden Markov Model with Measurement Noise Propagation

**DOI:** 10.1371/journal.pone.0140759

**Published:** 2015-10-16

**Authors:** Paddy J. Slator, Christopher W. Cairo, Nigel J. Burroughs

**Affiliations:** 1 Systems Biology Centre, University of Warwick, Coventry, United Kingdom; 2 Systems Biology Doctoral Training Centre, University of Warwick, Coventry, United Kingdom; 3 Department of Chemistry, University of Alberta, Edmonton, Alberta, Canada; J. Heyrovsky Institute of Physical Chemistry, CZECH REPUBLIC

## Abstract

We develop a Bayesian analysis framework to detect heterogeneity in the diffusive behaviour of single particle trajectories on cells, implementing model selection to classify trajectories as either consistent with Brownian motion or with a two-state (diffusion coefficient) switching model. The incorporation of localisation accuracy is essential, as otherwise false detection of switching within a trajectory was observed and diffusion coefficient estimates were inflated. Since our analysis is on a single trajectory basis, we are able to examine heterogeneity between trajectories in a quantitative manner. Applying our method to the lymphocyte function-associated antigen 1 (LFA-1) receptor tagged with latex beads (4 s trajectories at 1000 frames s^−1^), both intra- and inter-trajectory heterogeneity were detected; 12–26% of trajectories display clear switching between diffusive states dependent on condition, whilst the inter-trajectory variability is highly structured with the diffusion coefficients being related by *D*_1_ = 0.68*D*_0_ − 1.5 × 10^4^ nm^2^ s^−1^, suggestive that on these time scales we are detecting switching due to a single process. Further, the inter-trajectory variability of the diffusion coefficient estimates (1.6 × 10^2^ − 2.6 × 10^5^ nm^2^ s^−1^) is very much larger than the measurement uncertainty within trajectories, suggesting that LFA-1 aggregation and cytoskeletal interactions are significantly affecting mobility, whilst the timescales of these processes are distinctly different giving rise to inter- and intra-trajectory variability. There is also an ‘immobile’ state (defined as *D* < 3.0 × 10^3^ nm^2^ s^−1^) that is rarely involved in switching, immobility occurring with the highest frequency (47%) under T cell activation (phorbol-12-myristate-13-acetate (PMA) treatment) with enhanced cytoskeletal attachment (calpain inhibition). Such ‘immobile’ states frequently display slow linear drift, potentially reflecting binding to a dynamic actin cortex. Our methods allow significantly more information to be extracted from individual trajectories (ultimately limited by time resolution and time-series length), and allow statistical comparisons between trajectories thereby quantifying inter-trajectory heterogeneity. Such methods will be highly informative for the construction and fitting of molecule mobility models within membranes incorporating aggregation, binding to the cytoskeleton, or traversing membrane microdomains.

## Introduction

Single particle tracking (SPT), fluorescence recovery after photobleaching (FRAP), and fluorescence correlation spectroscopy (FCS) experiments have demonstrated that rather than moving freely, molecules in the plasma membrane tend to exhibit heterogenous motion. This heterogeneity occurs on a variety of scales, and a number of potential mechanisms have been proposed to explain the behaviour. These include: lipid microdomains [1, 2], compartmentalisation by the cytoskeleton (so-called ‘hop diffusion’) [3, 4], protein-protein interactions [5], and inhomogeneity in the plasma membrane environment [6]. There are a number of mechanistic models which reproduce anomalous diffusion [7]. Current thinking suggests that multiple mechanisms combine to form a hierarchical structure in the plasma membrane [8].

SPT experiments can directly observe the diffusion of lipids, proteins, and other complexes in the cell membrane, providing significant insight into membrane structure. In an SPT experiment the molecule of interest has an observable tag attached, allowing tracking of the tag’s 2D position over a number of time steps. Possible tags include a gold nanoparticle [9], a quantum dot [4], a fluorophore [10], or a latex bead [5]. Gold nanoparticle, quantum dot, and latex bead experiments can image the particle at high temporal resolution (up to 40000 frames s^−1^ [3]) over a long period (seconds). However, the tags are large relative to the molecules they label, with typical diameters of 10 nm for quantum dots [4]; 40 nm, gold nanoparticles [11]; 1000 nm, latex beads [5]. Other experiments have tracked single molecules by tagging with much smaller fluorophores but, due to photobleaching, can only track for much shorter periods [10], and thus provide shorter trajectories.

An open question is the extent to which the tracked tag represents the movement of the molecule of interest. General artifacts that may be associated with the use of a tag for SPT experiments include multivalent binding, drag, interaction with the extracellular matrix, and the binding of the label itself [12]. Additionally, experimental artifacts could result from movement of the particle out of the plane of focus or from the tracking algorithm which converts the raw video data to a trajectory. There is evidence that beads affect the estimated value of diffusion coefficients [13]. For example, results from gold nanoparticle experiments by the Kusumi lab report the presence of very fast diffusion within membrane compartments [9], much faster (by around a factor of ten) than in all other studies in the field. A possible explanation is that the nanoparticles used by Kusumi make the diffusion coefficient of the tag-target complex substantially different than that of the untagged molecule [4]. The fact that the tag is diffusing in solution whilst the molecule is in the membrane also causes a concern, potentially giving a weighted average of these two diffusion coefficients. Another possible cause of bead artifacts is crosslinking of proteins due to multivalent presentation. These issues highlight the importance of decoupling the particle behaviour from that of the tag, including dealing with experimental localisation error [14–16].

There are a number of techniques for analysing SPT data, including specific methods for the detection of deviations from free diffusion. The simplest and most common approach is to use a mean squared displacement (MSD) analysis. An unconfined random walk has a cumulative MSD that is linear as a function of time [17], whilst a negative deflection in MSD (anomalous diffusion) can be caused by movement in a confined environment, and a positive deflection suggests directed motion. MSD curves are often analysed for interpretable features such as the linear gradient (diffusion coefficient) and intercept (localisation accuracy); however, the subjectivity inherent in this approach has been suggested as the cause of discrepancies between studies [15]. Alternatively, theoretical MSD curves can be fitted to the data for various physical models (e.g. free diffusion, confined diffusion, hop diffusion, directed motion) [11, 18, 19]. Statistical analysis can be used to determine which theoretical model best describes the experimental MSD curve [4, 20]. However, these techniques are limited since they can only detect heterogeneity across multiple trajectories.

Methods for detecting heterogeneity within single trajectories (or ‘microheterogeneity’ [21]) have also been developed, most utilising statistics that detect deviations from random walk behaviour. For example, local periods of confinement can be detected by particles spending a significantly longer period of time within a fixed circle than a random walk [22–24], this has been utilised to detect trapping in experimental data [25–27]. This method has been further developed as a first passage time (FPT) analysis, which also gives an estimate of the size of confinement zones [21]. Other methods segment single trajectories based on transient changes in diffusion modalities, including detection of changes in the diffusion coefficient [28], local confinement and directed motion [29, 30]. Meilhac *et al*. [31] developed an algorithm which detects if a particle is moving between different confinement zones (i.e. exhibiting hop diffusion). The majority of these methods use generic properties of Brownian motion (random walks) to detect deviations, and thus, do not have an underlying mechanistic model. More information (with a corresponding increase in statistical power) can potentially be extracted by using a model that allows parametrisation of the heterogeneity and associated processes. Such models have been proposed in a hidden Markov model (HMM) framework. For instance, Das *et al*. developed a HMM for LFA-1 interacting with the actin cytoskeleton, where LFA-1 moves between “free” and “bound” states, moving with a different diffusion coefficient in each state [32]. Monnier developed a method which chooses between multiple modes of diffusion, such as directed motion, and diffusion with a variable diffusion coefficient [33, 34]. Persson *et al*. developed a HMM based method which takes multiple trajectories as input, and infers the number of diffusive states, the diffusion coefficients and the state transition rates [35].

Here we develop an improved single trajectory analysis, based on the two-state diffusion model of Das *et al*. [32]. We make two key changes to their analysis, firstly, we analyse each trajectory separately; the pooled analysis of [32] assumes homogeneity across trajectories, which we find is incorrect. This allows us to determine if individual trajectories have evidence for switching between two diffusive states, as opposed to remaining in one state throughout. Secondly, we allow for localisation accuracy. We demonstrate that a failure to do so can lead to the erroneous detection of a high degree of heterogeneity caused by structured measurement noise. We use a Bayesian analysis for both model parameter inference and model selection, using Markov chain Monte Carlo (MCMC) algorithms for both.

We apply our methods to previously published LFA-1 SPT data [5, 21, 32], LFA-1 being a cell membrane adhesion receptor on T cells that is known to interact with the cytoskeleton and exhibits multiple states with different diffusion properties, as shown by previous SPT analysis [5, 21, 32, 36]. LFA-1 has at least two affinity states, including a low affinity closed conformation and a high affinity open conformation, which are dependent on the cytoskeletal protein talin [37]. Activation of T cells, e.g. with phorbol-12-myristate-13-acetate (PMA), causes a number of changes in the behaviour of LFA-1, including a shift from the low to the high affinity state [38, 39] with an associated change in mobility [5, 40, 41]. The protease calpain releases LFA-1 from attachment to the cytoskeleton by cleaving the talin head domain [42]. By examining 4 treatments we find multiple modes of heterogeneity are present, including switching in the diffusion coefficient within single trajectories.

## Methods

Consider a single particle trajectory $X={\left\{\Delta X_{i},\Delta t_{i}\right\}}_{i=1}^{N}$ with displacements Δ*X*_*i*_ at discrete time points *i* = 1, 2…*N*, where Δ*X*_*i*_ = (Δ*X*_*i*1_, Δ*X*_*i*2_) is 2D. We aim to determine if a trajectory is consistent with a single diffusion process throughout, i.e. a one-state diffusion with diffusion coefficient *D* (to be determined), or if there is evidence of switching of the diffusion coefficient between two (again, to be determined) values, *D*_0_ and *D*_1_, i.e. a two-state diffusion model,

$$D_{0}\;\overset{p_{01}}{\underset{p_{10}}{⇌}}\;D_{1}$$ (1)

where *p*_01_, *p*_10_ are the probability of switching per frame. Both these models can be considered with or without measurement noise giving 4 models. Using a Bayesian methodology, for each model we developed an MCMC algorithm to sample the posterior distribution *π*(*θ*∣**X**, *M*_*i*_) of the model *M*_*i*_ and parameters *θ*, i.e. on individual trajectories we estimate the diffusion coefficient *D* for the one-state model, and the two diffusion coefficients *D*_0_, *D*_1_, with switching times between the two states for the two-state model. We also computed the marginal likelihood *π*(**X**∣*M*_*i*_) (either analytically, through MCMC sampling or, for the models with measurement noise, using an approximation). From the marginal likelihood we can compute the support for each model from the data, and thus determine the posterior model probability ratio $\pi \left(M_{1D}|X\right)/\pi \left(M_{2D}|X\right)$ for each trajectory. Under an equiprobable model prior this is equivalent to the Bayes factor $\pi \left(X|M_{1D}\right)/\pi \left(X|M_{2D}\right)$. These methods and associated algorithms are given here and in S1 Algorithms, but the Results can be read without this section.

### One-state diffusion model without measurement noise

For a particle diffusing with a diffusion coefficient *D*, the log likelihood of a trajectory **X** is

$$\log_{e}\pi \left(X|D\right)=\sum_{i=1}^{N}\log_{e}N\left(\Delta X_{i};0,2D\Delta t_{i}\right).$$ (2)

Here and throughout we use the same notation for a probability distribution and its (joint) pdf. We use a flat prior on *D*, *π*(*D*) = Unif(*D*; 0, *D*_*max*_), so the posterior is

$$\pi \left(1/D|X\right)\propto \text{Gamma}(1/D;N+1,\frac{1}{4}\sum_{i=1}^{N}\frac{\Delta X_{i}^{2}}{\Delta t_{i}})1_{\left[0,D_{max}\right]}\left(D\right)$$ (3)

where $1_{\left[0,D_{max}\right]}\left(D\right)=1$ if *D* ∈ (0, *D*_*max*_) and 0 otherwise. We use this notation for the indicator function throughout.

Appropriate statistics can be computed from this posterior, either analytically or using a rejection sampler. For a rejection sampler the update is

$$1/D∼{\text{Gamma}}_{T}\;(N+1,\frac{1}{4}\sum_{i=1}^{N}\frac{\Delta X_{i}^{2}}{\Delta t_{i}},1/D_{max},\infty )$$ (4)

where Gamma_*T*(*α*, *β*, *x*_*min*_, *x*_*max*_)_ denotes a truncated Gamma distribution with parameters *α* and *β*, truncated at *x*_*min*_ and *x*_*max*_. We sample *K* updates from this distribution to give samples ${\left\{D^{\left(k\right)}\right\}}_{k=1}^{K}$, an estimate for the diffusion coefficient is then the posterior mean $\hat{D}=\frac{1}{K}\sum_{k=1}^{K}D^{\left(k\right)}$.

The marginal likelihood for this model is

$$\pi \left(X|M_{1D}\right)=\int_{0}^{\infty }dD\;\pi \left(X|D,M_{1D}\right)\pi \left(D\right).$$ (5)

Changing variables from *D* to *D* ^−1^ and rearranging into a standard incomplete upper gamma function gives

$$\pi \left(X|M_{1D}\right)=\frac{1}{D_{max}}\;\prod_{i=1}^{N}\;\frac{1}{4\pi \Delta t_{i}}\;{\left(\sum_{i=1}^{N}\frac{\Delta X_{i}^{2}}{4\Delta t_{i}}\right)}^{1-N}\;\Gamma \left(N-1,\frac{1}{D_{max}}\sum_{i=1}^{N}\frac{\Delta X_{i}^{2}}{4\Delta t_{i}}\right).$$ (6)

where Γ is the upper incomplete Gamma function, see S1 Text.

### Two-state diffusion model without measurement noise

We use the hidden Markov model described by Das *et al*. [32] with four model parameters, *θ* = {*D*_0_, *D*_1_, *p*_01_, *p*_10_}, two diffusion coefficients *D*_0_, *D*_1_ and transition probabilities *p*_10_, *p*_01_ between the two hidden states. Denoting the hidden state by *z*_*i*_ at time frame *i*, the particle moves between *z*_*i*_ = 0 (diffusion with *D* = *D*_0_) and *z*_*i*_ = 1 (diffusion with *D* = *D*_1_) for *N* time steps, giving a trajectory **X** and hidden state sequence $z={\left\{z_{i}\right\}}_{i=1}^{N}$. The model can be written

$$z_{i}{|}_{z_{i-1}}∼\text{Bernoulli}\left(z_{i-1}\left(1-p_{10}\right)+\left(1-z_{i-1}\right)p_{01}\right),\Delta X_{i}{|}_{z_{i}}∼N\left(0,2D_{z_{i}}\Delta t_{i}\right).$$ (7)

We use conjugate priors, the full prior being

$$\pi \left(\theta \right)=\text{Unif}\left(D_{0};0,D_{max}\right)\text{Unif}\left(D_{1};0,D_{max}\right)\text{Beta}\left(p_{01};a_{0},b_{0}\right)\text{Beta}\left(p_{10};a_{1},b_{1}\right)$$ (8)

$$\pi \left(z_{1}|\theta \right)=\text{Bernoulli}\;\left(z_{1};\frac{p_{01}}{p_{10}+p_{01}}\right).$$ (9)

The prior on the initial state is the stationary distribution for the Markov chain. The posterior distribution is then given by,

$$\begin{array}{cc} \pi \left(\theta ,z|X\right) & \propto \pi \left(\theta \right)\pi \left(z_{1}|\theta \right)\prod_{i=1}^{N}N\;\left(\Delta X_{i};0,2D_{z_{i}}\Delta t_{i}\right)\\ & \times \prod_{i=2}^{N}\text{Bernoulli}\;(z_{i};z_{i-1}\left(1-p_{10}\right)+\left(1-z_{i-1}\right)p_{01}). \end{array}$$ (10)

We developed an MCMC algorithm to sample this posterior, specifically we can generate samples ${\left\{D_{0}^{\left(k\right)},D_{1}^{\left(k\right)},p_{01}^{\left(k\right)},p_{10}^{\left(k\right)},z^{\left(k\right)}\right\}}_{k=K_{B}}^{K}$ from the posterior distribution using a Gibbs sampler, see below and in S1 Algorithms as pseudocode. Here and throughout we denote the total number of MCMC steps *K* and the length of the burn-in *K*_*B*_. The mean of these posterior samples {$\hat{D_{0}}$, $\hat{D_{1}}$, $\hat{p_{01}}$, $\hat{p_{10}}$, $\hat{z}$} is an estimate for the parameters and hidden state sequence.

We sample from the posterior distribution Eq (10) by sampling sequentially from the conditional distributions. For *D*_0_ and *D*_1_ these are

$$\pi \left(D_{0}|D_{1},p_{01},p_{10},z,X\right)\propto \text{Unif}\left(D_{0};0,D_{max}\right)\prod_{z_{i}=0}N\;\left(\Delta X_{i};0,2D_{z_{i}}\Delta t_{i}\right)$$ (11)

$$\pi \left(D_{1}|D_{0},p_{01},p_{10},z,X\right)\propto \text{Unif}\left(D_{1};0,D_{max}\right)\prod_{z_{i}=1}N\;\left(\Delta X_{i};0,2D_{z_{i}}\Delta t_{i}\right).$$ (12)

Hence the updates are

$$D_{0}^{-1}∼{\text{Gamma}}_{T}\;(\eta_{0}+1,\sum_{z_{i}=0}\frac{\Delta X_{i}^{2}}{4\Delta t_{i}},\frac{1}{D_{max}},\infty )$$ (13)

$$D_{1}^{-1}∼{\text{Gamma}}_{T}\;(\eta_{1}+1,\sum_{z_{i}=1}\frac{\Delta X_{i}^{2}}{4\Delta t_{i}},\frac{1}{D_{max}},\infty )$$ (14)

where *η*_0_ = ∑_*z*_*i*_=0_1 and *η*_1_ = ∑_*z*_*i*_=1_1. We sample from the truncated distribution, by sampling from the full Gamma distribution, then resampling if *D*_0_ or *D*_1_ is bigger than *D*_*max*_. If *η*_0_ = 0 then $\sum_{z_{i}=0}\frac{\Delta X_{i}^{2}}{4\Delta t_{i}}=0$, and the Gamma distribution is undefined, so we sample *D*_0_ from the prior Unif(0, *D*_*max*_). If *η*_1_ = 0 we sample *D*_1_ from Unif(0, *D*_*max*_). For the transition probabilities let *n*_*jk*_ be the number of transitions from state *j* to state *k* in the hidden state sequence **z**, i.e.

$$n_{jk}=\sum_{{i|}_{z_{i}=j,z_{i+1}=k}}1.$$ (15)

Since we have chosen conjugate priors the updates are

$$p_{01}∼\text{Beta}\left(a_{0}+n_{01},b_{0}+n_{00}\right)$$ (16)

$$p_{10}∼\text{Beta}\left(a_{1}+n_{10},b_{1}+n_{11}\right).$$ (17)

The hidden state **z** is updated step by step. Since **z** is a Markov chain each *z*_*i*_ depends only on the neighbouring points *z*_*i*−1_ and *z*_*i*+1_, so the conditional distribution is

$$\begin{array}{cc} \pi \left(z_{i}|z_{i-1},z_{i+1},\theta ,X\right) & \propto \text{Bernoulli}\left(z_{i};z_{i-1}\left(1-p_{10}\right)+\left(1-z_{i-1}\right)p_{01}\right)\\ & \times N\left(\Delta X_{i};0,2D_{z_{i}}\Delta t_{i}\right)\\ & \times \text{Bernoulli}\left(z_{i+1};z_{i}\left(1-p_{10}\right)+\left(1-z_{i}\right)p_{01}\right). \end{array}$$ (18)

By normalising Eq (18) we can compute the probabilities *π*(*z*_*i*_∣*z*_*i*−1_, *z*_*i*+1_, *θ*, **X**) for *z*_*i*_ = 0, 1 which gives the update

$$z_{i}{|}_{\theta ,z_{i\pm 1}}∼\text{Bernoulli}\;(\pi (z_{i}=1|z_{i-1},z_{i+1},\theta ,X)).$$ (19)

The endpoint conditionals are slightly modified. For *i* = 1 and *i* = *N* we have

$$\pi \left(z_{1}|z_{2},\theta ,X\right)\propto N\;\left(\Delta X_{1};0,2D_{z_{1}}\Delta t_{1}\right)\text{Bernoulli}\;(z_{2};z_{1}\left(1-p_{10}\right)+\left(1-z_{1}\right)p_{01})$$ (20)

$$\pi \left(z_{N}|z_{N-1},\theta ,X\right)\propto \text{Bernoulli}\;(z_{N};z_{N-1}\left(1-p_{10}\right)+\left(1-z_{N-1}\right)p_{01})\;N\;\left(\Delta X_{N};0,2D_{z_{N}}\Delta t_{N}\right).$$ (21)

Thus, we can sequentially update **z** by updating each *z*_*i*_ for *i* = 1..*N*.

We also impose the condition *D*_0_ < *D*_1_, which we enforce after the MCMC run as follows: if the posterior means $\hat{D_{0}}>\hat{D_{1}}$ then we swap the *D*_0_, *D*_1_ chains, swap the *p*_01_, *p*_10_ chains, and swap the 0 and 1 states in the hidden state **z** throughout the run. This is possible because although state identity switching (0 ↔ 1) is possible because of a permutation symmetry during a run, it isn’t observed to occur.

There are a number of methods for estimating the marginal likelihood using MCMC sampling, including that of Chen [43], utilising a single MCMC chain, and Chib [44], requiring additional MCMC chains to be constructed. Typically we used both to check our algorithms, but present the simplest approach in any given case. For this model the conditional posterior *π*(*θ*∣**z**, **X**) is normalisable; Chen’s formula then reads

$$\pi \left(X|M_{2D}\right)=\log_{e}\pi \left(X|\theta^{*}\right)-\log_{e}[\frac{1}{K}\;\sum_{k=K_{B}}^{K}\frac{g(\theta^{\left(k\right)}|z^{\left(k\right)})}{\pi (\theta^{*})}\frac{\pi (\theta^{*}|z^{\left(k\right)},X)}{\pi (\theta^{\left(k\right)}|z^{\left(k\right)},X)}]$$ (22)

where $\theta^{*}=\left\{D_{0}^{*},D_{1}^{*},p_{01}^{*},p_{10}^{*}\right\}$ is a suitably chosen fixed point, such as the maximum likelihood, *θ* ^(*k*)^ and **z** ^(*k*)^ are samples from the MCMC run and *g*(*θ* ^(*k*)^∣**z** ^(*k*)^) is an arbitrary distribution, but its choice affects the variance of the estimate. If we choose *g*(*θ* ^(*k*)^∣**z** ^(*k*)^) = *π*(*θ* ^(*k*)^∣**z** ^(*k*)^, **X**) then we remove *θ* ^(*k*)^ from the right hand side, obtaining

$$\log_{e}\pi \left(X|M_{2D}\right)=\log_{e}\pi \left(X|\theta^{*}\right)-\log_{e}[\frac{1}{K}\;\sum_{k=K_{B}}^{K}\frac{\pi (\theta^{*}|z^{\left(k\right)},X)}{\pi (\theta^{*})}].$$ (23)

Thus, the sum runs over **z** ^(*k*)^, the MCMC samples, and for each **z** ^(*k*)^ we have to evaluate *π*(θ*∣**z** ^(*k*)^, **X**)/*π*(θ*). The log likelihood term, log_*e*_ *π*(**X**∣θ*), is calculated by the forward algorithm described in [32]. For the *π*(θ*∣**z** ^(*k*)^, **X**) term, we factorize

$$\begin{array}{cc} \pi \left(\theta^{*}|z^{\left(k\right)},X\right) & =\pi \left(D_{0}^{*}|z^{\left(k\right)},X\right)\pi \left(D_{1}^{*}|D_{0}^{*},z^{\left(k\right)},X\right)\pi \left(p_{01}^{*}|D_{0}^{*},D_{1}^{*},z^{\left(k\right)},X\right)\\ & \times \pi \left(p_{10}^{*}|p_{01}^{*},D_{0}^{*},D_{1}^{*},z^{\left(k\right)},X\right)\\ & =\pi \left(D_{0}^{*}|z^{\left(k\right)},X\right)\pi \left(D_{1}^{*}|z^{\left(k\right)},X\right)\pi \left(p_{01}^{*}|z^{\left(k\right)},X\right)\pi \left(p_{10}^{*}|z^{\left(k\right)},X\right) \end{array}$$ (24)

where the second line follows since the parameters are independent when **z** ^(*k*)^ is given.

The joint pdf is thus,

$$\begin{array}{cc} \pi \left(\theta^{*}|z^{\left(k\right)},X\right) & ={\text{Gamma}}_{T}\;(\frac{1}{D_{0}};\eta_{0}+1,\frac{1}{4}\sum_{z_{i}^{\left(k\right)}=0}\frac{\Delta X_{i}^{2}}{\Delta t_{i}},\frac{1}{D_{max}},\infty )\\ & \times {\text{Gamma}}_{T}\;(\frac{1}{D_{1}};\eta_{1}+1,\frac{1}{4}\sum_{z_{i}^{\left(k\right)}=1}\frac{\Delta X_{i}^{2}}{\Delta t_{i}},\frac{1}{D_{max}},\infty )\\ & \times \text{Beta}\left(p_{01};n_{01}+a_{0},n_{10}+b_{0}\right)\text{Beta}\left(p_{10};n_{10}+a_{1},n_{11}+b_{1}\right) \end{array}$$ (25)

at a given value θ*, where $\eta_{0}=\sum_{z_{i}^{\left(k\right)}=0}1$ and $\eta_{1}=\sum_{z_{i}^{\left(k\right)}=1}1$, and $z_{i}^{\left(k\right)}$ is the *i*th term in the sequence **z** ^(*k*)^. The normalisation term for the truncated distribution is $\Gamma {\left(\frac{1}{4D_{max}}\;\sum_{z_{i}^{\left(k\right)}=0}\frac{\Delta X_{i}^{2}}{\Delta t_{i}},\eta_{0}+1\right)}^{-1}$, where Γ is the upper incomplete gamma function. In practice, the normalisation factor is very close to 1, since the choice of *D*_*max*_ is sufficiently high.

Eq (25) is valid except when *η*_0_ = 0 or *η*_1_ = 0, in which case we have $\pi \left(D_{0}^{*}|z^{\left(k\right)},X\right)=\text{Unif}\left(D_{0};0,D_{max}\right)$ or $\pi \left(D_{1}^{*}|z^{\left(k\right)},X\right)=\text{Unif}\left(D_{1};0,D_{max}\right)$ respectively. The prior is

$$\pi \left(\theta^{*}\right)=\{\begin{array}{cc} \frac{D_{0}^{2}}{D_{max}}\frac{D_{1}^{2}}{D_{max}}\text{Beta}\left(p_{01};a_{0},b_{0}\right)\text{Beta}\left(p_{01};a_{1},b_{1}\right) & \text{if}\;\frac{1}{D_{0}},\frac{1}{D_{1}}\in \left[\frac{1}{D_{max}},\infty \right]\\ 0 & \text{otherwise} \end{array}$$ (26)

which is easy to evaluate for each **z** ^(*k*)^ from the MCMC output. Hence we can evaluate Eq (23).

### One-state diffusion model with measurement noise

We now add a localisation error to the previous one-state diffusion model. The true particle position is hidden and denoted *U*_*i*_, whilst the measured position is *U*_*i*_ up to a Gaussian noise with variance *σ* ^2^, assumed known. In discrete time the model is

$$\Delta U_{i}∼N\left(0,2D\Delta t_{i}\right),X_{i}{|}_{U_{i}}∼N\left(U_{i},\sigma^{2}\right)$$ (27)

where Δ*U*_*i*_ = *U*_*i*+1_ − *U*_*i*_. In order to develop an MCMC sampler for *π*(*D*, **U**∣**X**) we note that

$$\pi \left(D,U|X\right)\propto \pi \left(D,U_{1}\right)\prod_{i=1}^{N+1}N\left(X_{i};U_{i},\sigma^{2}\right)\prod_{i=1}^{N}N\left(\Delta U_{i};0,2D\Delta t_{i}\right).$$ (28)

We select a conjugate prior $\pi \left(D,U_{1}\right)=\text{Unif}\left(D;0,D_{max}\right)N\left(U_{1};\mu_{U_{1}},\sigma_{U}^{2}\right)$, so the updates for *D* and **U** are Gibbs moves. The update for *D* is

$$1/D∼{\text{Gamma}}_{T}\;(N+1,\frac{1}{4}\;\sum_{i=1}^{N}\frac{\Delta U_{i}^{2}}{\Delta t_{i}},1/D_{max},\infty ).$$ (29)

The conditional distribution for *U*_*i*_ is a bridging distribution

$$\begin{array}{cc} \pi \left(U_{i}|U_{i-1},U_{i+1},D_{0},D_{1},p_{01},p_{10}\right) & \propto N\;\left(\Delta U_{i-1};0,2D\Delta t_{i-1}+2\sigma^{2}\right)\\ & \times N\;\left(\Delta U_{i};0,2D\Delta t_{i}+2\sigma^{2}\right)N\left(U_{i};X_{i},\sigma^{2}\right) \end{array}$$ (30)

comprising a product of three Gaussians. The update is thus,

$$U_{i}{|}_{D,U_{i\pm 1}}∼N\;\left(\mu_{i},1/\tau_{i}\right)$$ (31)

where, for *i* = 2 to *i* = *N* the precision and mean are

$$\tau_{i}=\frac{1}{2D\Delta t_{i-1}}+\frac{1}{2D\Delta t_{i}}+\frac{1}{\sigma^{2}},\mu_{i}=(\frac{U_{i-1}}{2D\Delta t_{i-1}}+\frac{U_{i+1}}{2D\Delta t_{i}}+\frac{X_{i}}{\sigma^{2}})\;\tau_{i}^{-1}$$ (32)

at the endpoints *i* = 1 and *i* = *N* + 1

$$\tau_{1}=\frac{1}{2D\Delta t_{1}}+\frac{1}{\sigma_{U}^{2}},\mu_{1}=(\frac{U_{2}}{2D\Delta t_{1}}+\frac{\mu_{U_{1}}}{\sigma_{U}^{2}})\;\tau_{1}^{-1}$$ (33)

$$\tau_{N+1}=\frac{1}{2D\Delta t_{N}}+\frac{1}{\sigma^{2}},\mu_{N+1}=(\frac{U_{N}}{2D\Delta t_{N}}+\frac{X_{N}}{\sigma^{2}})\;\tau_{N+1}^{-1}.$$ (34)

We used an approximation to compute the marginal likelihood; this involves ignoring the covariance between the displacements Δ*X*_*i*_ ∼ *N*(Δ*U*_*i*_, 2*σ* ^2^) and Δ*X*_*i*+1_ ∼ *N*(Δ*U*_*i*+1_, 2*σ* ^2^) that arises because of the common measurement error *X*_*i*_ − *U*_*i*_ at time point *i*. In this case the hidden variables *U*_*i*_ integrate out to give the posterior

$$\pi \left(D|X\right)\propto \pi \left(D\right)\prod_{i=1}^{N}N\;(\Delta X_{i};0,2D\Delta t_{i}+2\sigma^{2}).$$ (35)

We modified the previous one-state MCMC sampler to sample from this distribution, detailed in S1 Text and S1 Algorithms as pseudocode.

The sampler has a single Metropolis-Hastings move, so we calculate the marginal likelihood directly from the MCMC output, as described by Chib [45]. The log marginal identity is

$$\log_{e}\hat{\pi }\left(X|M_{1D}\right)=\log_{e}\pi \left(X|D^{*}\right)+\log_{e}\pi \left(D^{*}\right)-\log_{e}\hat{\pi }\left(D^{*}|X\right)$$ (36)

where we take $D^{*}=\hat{D}$, the mean of the posterior samples. We can evaluate log_*e*_ *π*(**X**∣D*) and log_*e*_ *π*(D*) easily. We can write log_*e*_ *π*(D*∣**X**) as [45]

$$\log_{e}\pi \left(D^{*}|X\right)=\log_{e}[\frac{E_{1}\;[\alpha \left(D\to D^{*}\right)q\left(D\to D^{*}\right)]}{E_{2}\;[\alpha (D^{*}\to D)]}]$$ (37)

where $E_{1}$ is with respect to *π*(*D*∣**X**) and $E_{2}$ is with respect to *q*(D* → *D*). From the MCMC output we have *K* − *K*_*B*_ samples from the posterior distribution *π*(*D*∣**X**), ${\left\{D^{\left(k\right)}\right\}}_{K=K_{B}}^{K}$. We then simulate *K* − *K*_*B*_ samples from the proposal distribution *q*(D* → *D*) ∼ *N*(D*, *S*_*D*_), giving ${\left\{{\tilde{D}}^{\left(j\right)}\right\}}_{j=1}^{K-K_{B}}$. An estimate for log_*e*_ *π*(D*∣**X**) is then

$$\log_{e}\hat{\pi }\left(D^{*}|X\right)=\log_{e}[\frac{{\left(K-K_{B}\right)}^{-1}\;\sum_{k=K_{B}}^{K}\alpha \left(D^{\left(k\right)}\to D^{*}\right)q\left(D^{\left(k\right)}\to D^{*}\right)}{{\left(K-K_{B}\right)}^{-1}\;\sum_{j=1}^{K-K_{B}}\alpha \left(D^{*}\to {\tilde{D}}^{\left(j\right)}\right)}].$$ (38)

Hence we can calculate $\hat{\pi }\left(X|M_{1D}\right)$ using Eq (36).

### Two-state diffusion model with measurement noise

We now add a localisation error to the previous two-state diffusion hidden Markov model. Again, the true position is hidden and denoted *U*_*i*_. The model is given by,

$$\begin{array}{cc} z_{i}{|}_{z_{i-1}} & ∼\text{Bernoulli}\left(z_{i-1}\left(1-p_{10}\right)+\left(1-z_{i-1}\right)p_{01}\right),\\ \Delta U_{i}{|}_{z_{i}} & ∼N\left(0,2D_{z_{i}}\Delta t_{i}\right),\\ X_{i}{|}_{U_{i}} & ∼N\left(U_{i},\sigma^{2}\right) \end{array}$$ (39)

i.e. there is both a continuous hidden state *U*_*i*_ and a discrete hidden state *z*_*i*_. We developed an MCMC algorithm which samples from the full conditional distribution *π*(*θ*, **U**, **z**∣**X**). Let *θ* = {*D*_0_, *D*_1_, *p*_01_, *p*_10_}, the posterior for this model is (Δ*U*_*i*_ = *U*_*i*+1_ − *U*_*i*_)

$$\begin{array}{cc} \pi \left(\theta ,U,z|X\right) & \propto \pi \left(\theta ,U_{1},z_{1}\right)\prod_{i=1}^{N+1}N\;\left(X_{i};U_{i},\sigma^{2}\right)\prod_{i=1}^{N}N\;\left(\Delta U_{i};0,2D_{z_{i}}\Delta t_{i}\right)\\ & \times \prod_{i=1}^{N-1}\text{Bernoulli}\left(z_{i+1};z_{i}\left(1-p_{10}\right)+\left(1-z_{i}\right)p_{01}\right). \end{array}$$ (40)

The priors on *θ* and *z*_1_ are the same as the two-state diffusion model without measurement noise, given in Eq (8), and we use a normal prior (with mean *μ*_*U*_1__, variance $\sigma_{U}^{2}$) on *U*_1_. The full prior is then

$$\begin{array}{cc} \pi \left(\theta ,U_{1},z_{1}\right) & =\text{Unif}\left(D_{0};0,D_{max}\right)\text{Unif}\left(D_{1};0,D_{max}\right)\text{Beta}\left(p_{01};a_{0},b_{0}\right)\text{Beta}\left(p_{10};a_{1},b_{1}\right)\\ & \times N\left(U_{1};\mu_{U_{1}},\sigma_{U}^{2}\right)\text{Bernoulli}\;(z_{1};\frac{p_{01}}{p_{10}+p_{01}}). \end{array}$$ (41)

The MCMC updates are mostly identical to the two-state diffusion model without measurement noise, but with the observed displacements Δ*X*_*i*_ replaced by the hidden state displacements Δ*U*_*i*_. Thus, for *D*_0_ and *D*_1_ we have

$$1/D_{0}∼{\text{Gamma}}_{T}\;(\eta_{0}+1,\frac{1}{4}\sum_{z_{i}=0}\frac{\Delta U_{i}^{2}}{\Delta t_{i}},1/D_{max},\infty )$$ (42)

$$1/D_{1}∼{\text{Gamma}}_{T}\;(\eta_{1}+1,\frac{1}{4}\sum_{z_{i}=1}\frac{\Delta U_{i}^{2}}{\Delta t_{i}},1/D_{max},\infty )$$ (43)

where *η*_0_ = ∑_*z*_*i*_=0_1 and *η*_1_ = ∑_*z*_*i*_=1_1 as before. Similarly, in the **z** update we substitute Δ*X*_*i*_ for Δ*U*_*i*_ in Eqs (18), (20) and (21),

$$\begin{array}{cc} \pi \left(z_{i}|z_{i-1},z_{i+1},\theta ,U\right) & \propto \text{Bernoulli}\left(z_{i};z_{i-1}\left(1-p_{10}\right)+\left(1-z_{i-1}\right)p_{01}\right)\times N\left(\Delta U_{i};0,2D_{z_{i}}\Delta t_{i}\right)\\ & \times \text{Bernoulli}\left(z_{i+1};z_{i}\left(1-p_{10}\right)+\left(1-z_{i}\right)p_{01}\right). \end{array}$$ (44)

$$\pi \left(z_{1}|z_{2},\theta ,U\right)\propto N\left(\Delta U_{1};0,2D_{z_{1}}\Delta t_{1}\right)\text{Bernoulli}(z_{2};z_{1}\left(1-p_{10}\right)+\left(1-z_{1}\right)p_{01})$$ (45)

$$\pi \left(z_{N}|z_{N-1},\theta ,U\right)\propto \text{Bernoulli}(z_{N};z_{N-1}\left(1-p_{10}\right)+\left(1-z_{N-1}\right)p_{01})\;N\;\left(\Delta U_{N};0,2D_{z_{N}}\Delta t_{N}\right).$$ (46)

The transition probability updates are identical to Eqs (16) and (17). The update for *U* is almost the same as the one-state diffusion model with measurement noise. We have a Gibbs update

$$U_{i}{|}_{\theta ,z,U_{i\pm 1}}∼N\;\left(\mu_{i},1/\tau_{i}\right)$$ (47)

where, for *i* = 2 to *i* = *N*, the precision and mean are

$$\tau_{i}=\frac{1}{2D_{z_{i-1}}\Delta t_{i-1}}+\frac{1}{2D_{z_{i}}\Delta t_{i}}+\frac{1}{\sigma^{2}},\mu_{i}=(\frac{U_{i-1}}{2D_{z_{i-1}}\Delta t_{i-1}}+\frac{U_{i+1}}{2D_{z_{i}}\Delta t_{i}}+\frac{X_{i}}{\sigma^{2}})\;\tau_{i}^{-1}$$ (48)

at the endpoints *i* = 1 and *i* = *N* + 1

$$\tau_{1}=\frac{1}{2D_{z_{1}}\Delta t_{1}}+\frac{1}{\sigma_{U}^{2}},\mu_{1}=(\frac{U_{2}}{2D_{z_{1}}\Delta t_{1}}+\frac{X_{1}}{\sigma_{U}^{2}})\;\tau_{1}^{-1}$$ (49)

$$\tau_{N+1}=\frac{1}{2D_{z_{N}}\Delta t_{N}}+\frac{1}{\sigma^{2}},\mu_{N+1}=(\frac{U_{N}}{2D_{z_{N}}\Delta t_{N}}+\frac{X_{N}}{\sigma^{2}})\;\tau_{N+1}^{-1}.$$ (50)

The MCMC updates for this model are given in pseudocode in S1 Algorithms.

To compute the marginal likelihood we used the same approximation as the one-state diffusion model with measurement noise, ignoring the covariance between the displacements Δ*X*_*i*_ ∼ *N*(Δ*U*_*i*_, 2*σ* ^2^) and Δ*X*_*i*+1_ ∼ *N*(Δ*U*_*i*+1_, 2*σ* ^2^). We failed to find an algorithm that could integrate over both hidden states (*U*_*i*_ and *z*_*i*_) to allow the (exact) marginal likelihood *π*(**X**∣*M*_2*D*_) to be computed. In this case the hidden variables *U*_*i*_ integrate out to give a posterior

$$\begin{array}{cc} \pi \left(\theta ,z|X\right) & \propto \pi \left(\theta \right)\pi \left(z_{1}|\theta \right)\prod_{i=1}^{N}N\;\left(\Delta X_{i};0,2\left(D_{z_{i}}\Delta t_{i}+\sigma^{2}\right)\right)\\ & \times \prod_{i=1}^{N-1}\text{Bernoulli}\left(z_{i+1};z_{i}\left(1-p_{10}\right)+\left(1-z_{i}\right)p_{01}\right). \end{array}$$ (51)

We modified the two-state diffusion model sampler to incorporate the *σ* ^2^ terms, see S1 Text and pseudocode in S1 Algorithms. This sampler can be used with the method of Chen, rewriting Chen’s formula as

$$\log_{e}\pi \left(X|M_{2D}\right)=\log_{e}\pi \left(X|\theta^{*}\right)-\log_{e}[\frac{1}{K}\;\sum_{k=1}^{K}\frac{g(\theta_{k}|z^{\left(k\right)})}{\pi (\theta^{\left(k\right)})}\frac{\pi (X|\theta^{*},z^{\left(k\right)})}{\pi (X|\theta^{\left(k\right)},z^{\left(k\right)})}\frac{\pi (z^{\left(k\right)}|\theta^{*})}{\pi (z^{\left(k\right)}|\theta^{\left(k\right)})}]$$ (52)

where *g* is any density function, *θ* ^(*k*)^, **z** ^(*k*)^ are samples from the posterior distribution, and θ* is a point of high density. If we choose *g* = *π*(*θ* ^(*k*)^), then an estimate for the marginal likelihood is

$$\begin{array}{cc} & \log_{e}\hat{\pi }\left(X|M_{2D}\right)=\log_{e}\left(X|\theta^{*}\right)-\;\log_{e}[\frac{1}{K}\;\sum_{k=1}^{K}\frac{\prod_{i=1}^{N}N\;\left(\Delta X_{i}^{2};0,2D_{z_{i}^{\left(k\right)}}^{*}\Delta t_{i}+2\sigma^{2}\right)}{\prod_{i=1}^{N}N\;\left(\Delta X_{i}^{2};0,2D_{z_{i}^{\left(k\right)}}^{\left(k\right)}\Delta t_{i}+2\sigma^{2}\right)}\\ & \times \frac{\text{Beta}\;(p_{01}^{*};n_{01}+1,n_{00}+1)\;\text{Beta}\;(p_{10}^{*};n_{10}+1,n_{11}+1)}{\text{Beta}\;(p_{01}^{\left(k\right)};n_{01}+1,n_{00}+1)\;\text{Beta}\;(p_{10}^{\left(k\right)};n_{10}+1,n_{11}+1)}]. \end{array}$$ (53)

The log likelihood, log_*e*_ *π*(**X**∣θ*), is calculated using a forward algorithm (S1 Text and pseudocode in S1 Algorithms). By computation of the marginal on multiple chains we found that its variance was small despite using the prior for the distribution *g*, (relative sd < 0.0001%). Chib’s method on selected trajectories also gave the same answer.

### Priors

In all algorithms we use weak priors. Specifically *D* ∼ Unif(0, *D*_*max*_ = 10^6^nm^2^s^−1)^ for the one-state diffusion model, and additionally $U_{1}\sim N\;\left(\mu_{U_{1}}=\left[\begin{array}{c} 0\\ 0 \end{array}\right],\sigma_{U}^{2}=\left[\begin{array}{cc} 10^{6}{\text{nm}}^{2} & 0\\ 0 & 10^{6}{\text{nm}}^{2} \end{array}\right]\right)$ for the one-state diffusion model with measurement noise. For the two-state diffusion model we use: *D*_0_, *D*_1_ ∼ Unif(0, *D*_*max*_ = 10^6^nm^2^s^−1)^, *p*_10_, *p*_01_ ∼ Beta(1, 1), with an initial (*i* = 1) hidden state, $z_{1}∼\text{Bernoulli}\left(\frac{p_{01}}{p_{01}+p_{10}}\right)$. Additionally, we used $U_{1}\sim N\;\left(\mu_{U_{1}}=\left[\begin{array}{c} 0\\ 0 \end{array}\right],\sigma_{U}^{2}=\left[\begin{array}{cc} 10^{6}{\text{nm}}^{2} & 0\\ 0 & 10^{6}{\text{nm}}^{2} \end{array}\right]\right)$ for the two-state diffusion model with measurement noise.

### Convergence of MCMC runs

To assess the convergence of the two-state diffusion model with measurement noise we used a multiple chain convergence diagnostic [46], specifically 12 chains with overdispersed initial values. We initialised *D*_0_ and *D*_1_ by sampling values *u*_0_, *u*_1_ from Beta(0.1, 0.1), then setting *D*_0_ = *u*_0_ *D*_*max*_ and *D*_1_ = *u*_1_ *D*_*max*_. The transition probabilities *p*_01_ and *p*_10_ were initialised from Beta(1, 1). The hidden state **z** was initialised by simulating a Markov chain using the initial transition probabilities *p*_01_ and *p*_10_. The initial value of **U** was set to the observed trajectory ${\left\{X_{i}\right\}}_{i=1}^{N+1}$. We considered the chains converged when the Gelman-Rubin diagnostic for each parameter was less than 1.1 [47, 48].

### Model selection between one-state and two-state diffusion models

We can calculate the log marginal likelihoods to compare the evidence for the one-state and two-state diffusion models; this can be done with or without measurement noise. Hence for each case we can calculate the log (base *e*) Bayes factor, log_*e*_ *B*_1*D*, 2*D*_ = log_*e*_ *π*(**X**∣*M*_1*D*_)) − log_*e*_ *π*(**X**∣*M*_2*D*_). The extent to which a model is supported by the evidence (i.e. the observed trajectory **X**) can then be assessed using a standard table such as in Kass *et al*., where a log Bayes factor of 3 is considered “strong” evidence for the relevant model [49]. We hence consider a value log_*e*_ *B*_1*D*, 2*D*_ > 3 as preference for a one-state diffusion model, and log_*e*_ *B*_1*D*, 2*D*_ < −3 as preference for a two-state diffusion model. The remaining trajectories (where −3 < log_*e*_ *B*_1*D*, 2*D*_ < 3) have no strong preference for either model.

## Results

Given a 2D trajectory, **X**, we developed MCMC algorithms (both with and without measurement noise) for inferring the posterior distribution of the parameters and hidden states of a two-state diffusion process, *π*(*θ*, **z**∣**X**). The parameters, *θ* = {*D*_0_, *D*_1_, *p*_01_, *p*_10_}, are the diffusion coefficients and frame transition probabilities, Eq (1), and **z** is the sequence of the inferred hidden diffusion state. Allowing for measurement noise propagates that uncertainty to the parameter estimates. We tested our algorithms on simulated data; S1 Fig shows an MCMC run of the two-state model with measurement noise, demonstrating accurate reconstruction of the parameters and hidden states. We also tested the sensitivity of the method to closely matched diffusion states, S2 Fig. The two-state model with measurement noise algorithm can accurately detect switching between regimes where diffusion coefficients differ by a factor of 1.5 (trajectory parameters set to those typical for the LFA-1 data). To determine whether the trajectory is better explained by this two-state model or a one-state diffusion (single diffusion coefficient *D*) we used the marginals *π*(**X**∣*M*); however, this proved difficult to calculate in our hands for the two-state model (with measurement noise). Therefore, we used an approximate likelihood (where the covariance between consecutive displacements is ignored, essentially a low measurement noise limit) where the marginals are computable for both the one-state and two-state models, see Methods. We used the Bayes factor of this approximation to determine if the two-state model is supported by the data more than a one-state diffusion process. We tested the model selection between the approximate one-state and two-state diffusion models with measurement noise; both on trajectories simulated from the full measurement noise model, and trajectories simulated without noise, S3 Fig. The model was able to successfully discriminate between one-state and two-state simulations, with a very low false positive rate when the diffusion coefficients were separated by a factor of 5 (0.005%, using log_*e*_ Bayes factor equal to ±3 as the threshold for model preference, see Methods). When separated by a factor of 2.5 there is a bias towards the one-state model, especially on two-state model simulations without measurement noise, S3B Fig. Thus, we may fail to detect some switching events between close diffusion coefficients, underestimating the number of trajectories preferring a two-state model.

We used our algorithms to analyse SPT data sets for the LFA-1 receptor on Jurkat T cells (4 s trajectories at 1000 frames s^−1^ [5]). The receptor was tagged with 1000 nm latex beads coated with the LFA-1 binding antibody TS-1/18. This dataset has been analysed previously [5, 21, 32] demonstrating that LFA-1 diffusion is heterogeneous. We applied our methods to four treatments: control (DMSO), treated with cytochalasin D (Cyto D), treated with phorbol-12-myristate-13-acetate (PMA), and treated with PMA with calpain inhibition (PMA+Cal-I). Cytochalasin D is an inhibitor of actin polymerisation, so effects due to the cytoskeleton should be decreased, PMA is a T cell activator, moving LFA-1 to the high affinity conformational state, and calpain releases LFA-1 from attachment to the cytoskeleton by cleaving the talin head domain [42]. Thus, the first two treatments explore the effect of the cytoskeleton on the predominantly low affinity LFA-1. PMA examines dynamics of high affinity LFA-1, whilst PMA+Cal-I examines high affinity LFA-1 under conditions of enhanced interaction with the cytoskeleton.

To determine the measurement accuracy, and whether measurement noise had to be incorporated into the model, we examined stationary beads (3 trajectories were available). These beads are attached to the surface and thus represent thermal motion and instrument noise. These beads are effectively stuck in a potential well and their movement is expected to be temporally homogeneous; thus no two-state diffusion structure should be detected. As presented below, we find that this is not the case unless a Gaussian measurement noise is incorporated into the inference. Therefore, throughout we present the analysis of LFA-1 trajectory data using the measurement noise models, comparing between the one-state diffusion and two-state diffusion model in the presence of noise. We use the approximate likelihood models for model discrimination only; all inferred parameters refer to the exact models.

### Stationary bead analysis to determine measurement accuracy and the importance of propagating measurement noise

Trajectories of stationary beads (immobilised on glass using cell-tak, imaged using the same set up as the LFA-1 data [5]) were used to determine the signal to noise ratio (S/N) and to estimate the measurement noise (*σ* ^2^). For each trajectory (2 s at 1000 frames s^−1^) we calculated the variance of individual displacements ${\left\{\Delta X_{i}\right\}}_{i=1}^{N}$ for both x and y directions, giving 6 estimates for the localisation accuracy (29.09 nm^2^, 23.55 nm^2^, 65.01 nm^2^, 39.31 nm^2^, 30.27 nm^2^, 59.41 nm^2^). This gives a mean *σ* ^2^ = 41.09 nm^2^ which we used as an estimate of the localisation accuracy throughout. The variance of individual displacements, Δ*X*_*i*_ for the LFA-1 data are: DMSO, 133.5 nm^2^ (giving S/N 3.25); Cyto D, 133.7 nm^2^ (S/N 3.25); PMA, 135.1 nm^2^ (S/N 3.29); PMA+Cal-I, 89.4 nm^2^ (S/N 2.18), indicating that signal is present in these displacements at this resolution.

**Fig 1:**
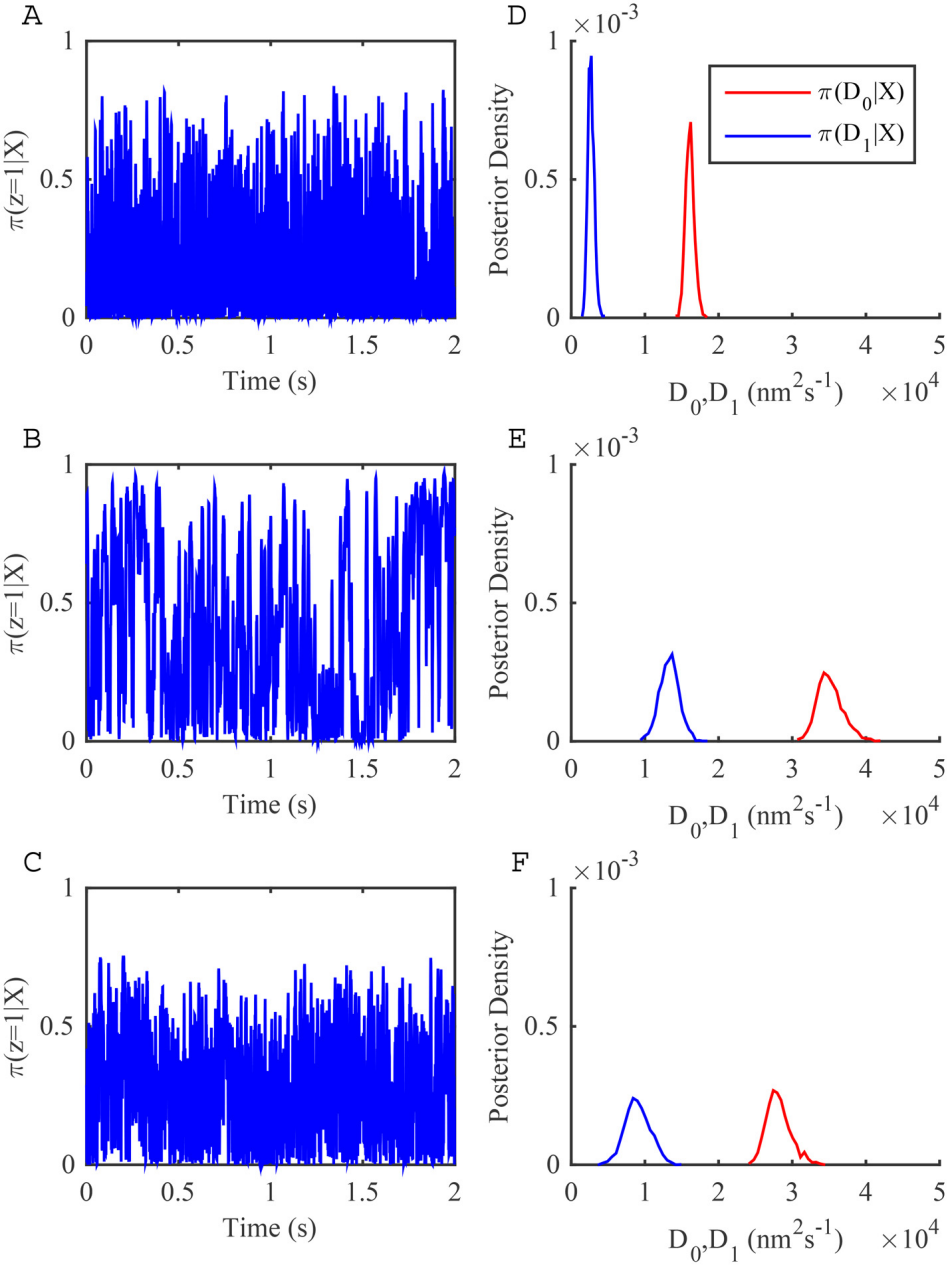
Fit of a two-state diffusion model without measurement noise to three stationary latex bead trajectories. MCMC output from chains of 20000 MCMC steps with a 10000 step burn-in. (A-C) Inference of the hidden state **z** shown as the probability of being in the low diffusion state. (D-F) Posterior distributions for the two diffusion coefficients: *D*_0_ (red) and *D*_1_ (blue). See Methods for priors and initial conditions.

**Fig 2:**
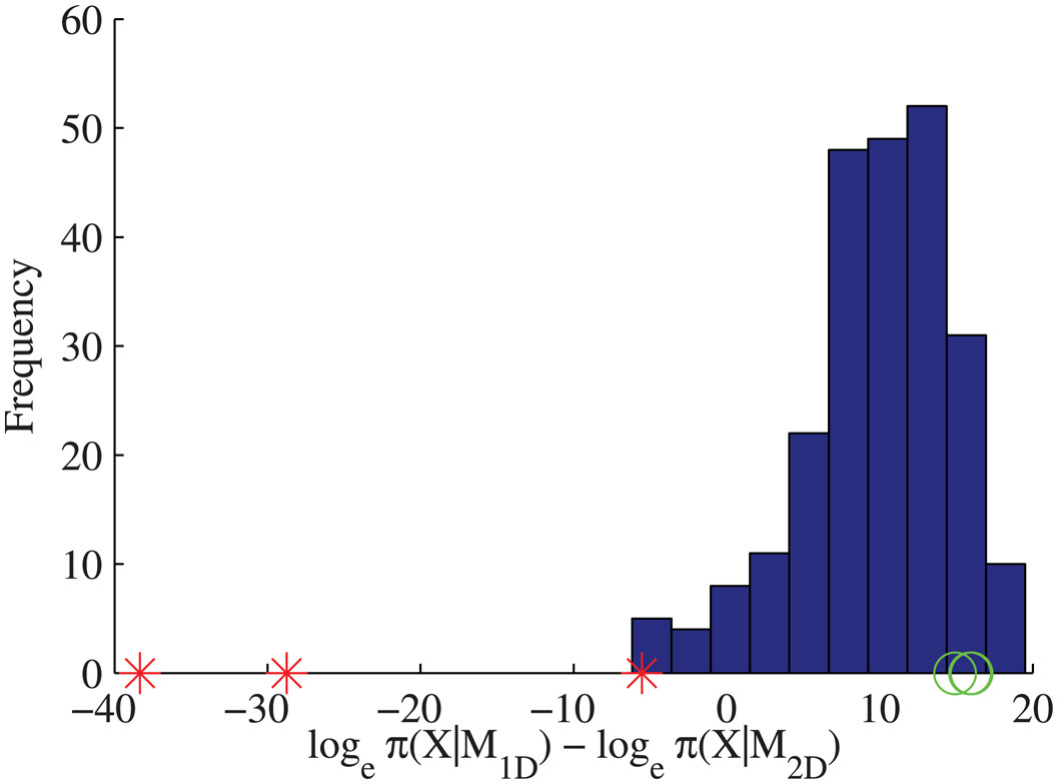
Model selection for one-state and two-state diffusion models on simulated stationary beads and stationary latex bead trajectories. Blue bars: Bayes factors from model selection on simulated stationary beads (*n* = 240) with added Gaussian noise (*σ* ^2^ = 41.09nm^2^). Single data points on axis: Bayes factors from model selection on stationary latex bead trajectories, both without (red asterisks) and with (green circles, *σ* ^2^ = 41.09nm^2^) measurement noise incorporated into the inference algorithm. Priors, see Methods.

The stationary beads also provide an opportunity to check that the measurement noise does not affect model selection: stationary beads should prefer a one-state diffusion model since the time series is homogeneous. If the two-state diffusion model is preferred then measurement noise, the tracking algorithm, or instrument noise contributes to the heterogeneity in the trajectory. We applied the one-state and two-state diffusion model algorithms (without measurement noise) to the three stationary beads. The two-state diffusion model showed high frequency switching behaviour (Fig 1A–1C), with two distinct (well separated) diffusion coefficients, (Fig 1D–1F). Crucially, the two-state diffusion model is strongly preferred for all 3 trajectories (Fig 2, red asterisks). Therefore there is evidence that tracked bead displacements are not unstructured and an analysis of LFA-1 trajectories using the models without allowing for measurement noise may be unreliable, due to this inherent inhomogeneity.

The stationary bead data were then analysed with the approximate one-state and two-state diffusion models with measurement noise using the estimated noise variance *σ* ^2^ = 41.09nm^2^ (recall the approximate models ignore the covariance between displacements since the marginal cannot be calculated for the full model). The incorporation of localisation accuracy eliminates the previous preference for a two-state diffusion model (Fig 2, green circles); preference for the one-state diffusion model is in fact very strong. We also tested whether Gaussian noise can cause deterioration of the model selection accuracy. We tested the model selection analysis without measurement noise on a set (*n* = 240) of simulated stationary bead trajectories with added Gaussian noise (the localisation error in the simulations was set to 41.09nm^2^). The model selection has a very strong preference for the one-state diffusion model (Fig 2, blue bars) so Gaussian noise alone causes a low false detection rate; the noise in the beads cannot therefore be Gaussian and/or independent.

There are two important conclusions: firstly, as pointed out by Michalet [15], the localisation accuracy is a potential source of bias, particularly for low signal to noise ratios, as found here. Thus, in any SPT analysis the measurement accuracy should be separately determined and an assessment made as to whether it affects the results. Secondly, the noise of the stationary beads does not appear to be independent, having a temporal correlation. Thus, the localisation accuracy is likely not constant along a trajectory. However, as shown here, incorporating Gaussian measurement noise into the model inference removes the erroneous preference for the two-state model for the stationary beads.

### Analysis of LFA-1 data: evidence of multiple diffusion states

**Fig 3:**
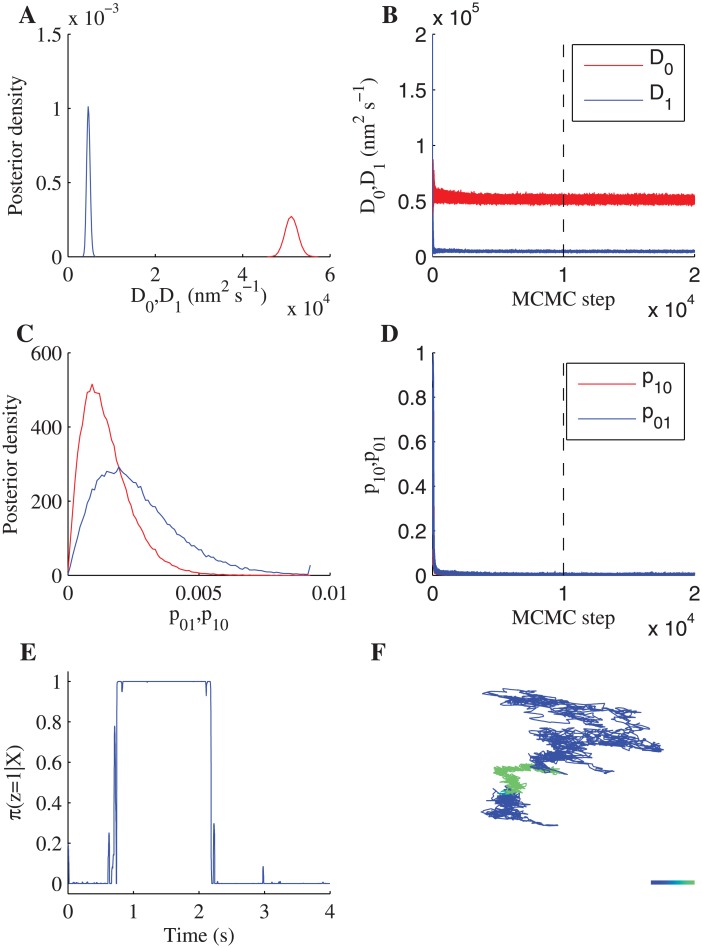
Fit of a two-state diffusion model with measurement noise to an LFA-1 trajectory (PMA+Cal-I treatment). MCMC output (12 independent chains of 20000 MCMC steps with a 10000 step burn-in). (A) The posteriors for the two diffusion coefficients, (B) corresponding samples (12 chains plotted in the same colour) for *D*_0_ (red) and *D*_1_ (blue) including burn-in (dashed line). (C) Posteriors for the switching probabilities per frame, (D) corresponding samples (12 chains) for *p*_01_ (red) and *p*_10_ (blue) including burn-in (dashed line). (E) State inference shown as the probability of being in the low diffusion state. (F) Trajectory coloured by the probability of being in the low diffusion state. Colour scale represents *π*(**z** = 1∣**X**) from 0 (blue, high diffusion state) to 1 (green, low diffusion state). Colorbar length: 100nm. Priors, see Methods.

We fitted the one-state and two-state diffusion models with measurement noise to each trajectory in the four treatments (36–75 trajectories depending on treatment, 4 s trajectories at 1000 frames s^−1^), thereby estimating parameters for these models for each trajectory. Convergence was confirmed using a multiple chain protocol, see Methods. An example of a fit to the two-state diffusion model with measurement noise is shown in Fig 3; inference of the hidden state shows clear evidence of state switching in this trajectory with a high probability of being in one or other of the two diffusion states and tight switching times. There is a large separation in the posterior distributions for the low and high diffusion coefficients, with the ratio of the posterior mean estimates being around 10.

**Fig 4:**
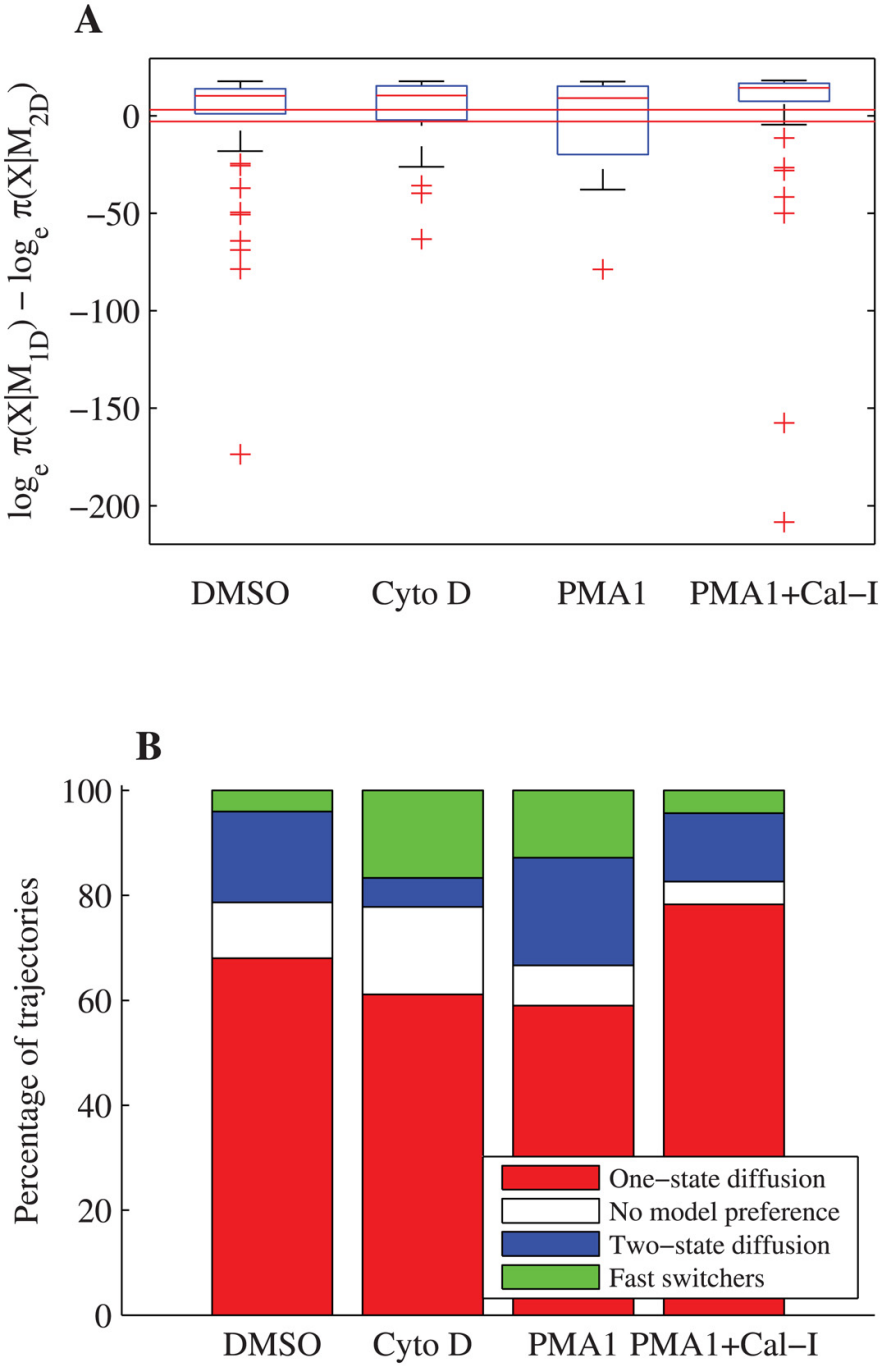
Model selection between approximate one-state and two-state diffusion models with measurement noise on LFA-1 trajectories. (A) Box and whisker plot of log Bayes factors by treatment, trajectories with log Bayes factor outside 1.5 times IQR are plotted as outliers (red crosses). The thresholds ±3 (red lines) are shown. (B) Stacked bar plot showing proportions for each preferred model and trajectories which demonstrate fast switching between diffusive states. A log Bayes factor of ±3 ((A), red lines) is considered preference for the relevant model. MCMC runs comprise 12 parallel chains of 20000 steps with a 10000 step burn-in. Priors, see Methods.

By calculating the marginal likelihood for the approximate one-state and two-state diffusion models with measurement noise, and hence the Bayes factor $B_{1D,2D}=\frac{\pi (X|M_{1D})}{\pi (X|M_{2D})}$, we then ascertained for each trajectory the evidence for a two-state compared to a one-state diffusion process. As described in Methods, we used fairly stringent criteria: if the log (base *e*) Bayes factor is smaller than -3 then we consider this preference for the two-state diffusion model, and greater than 3 as preference for the one-state diffusion model [49]. The number of trajectories with preference for each model was robust to the choice of Bayes factor threshold (S1 Table). Fig 4 shows the Bayes factor estimates for each condition, and the number of trajectories which preferred each model, grouped by treatment. There are a total of 16 DMSO (out of a total of 75, 21%), 8 Cyto D (out of 36, 22%), 13 PMA (out of 19, 33%) and 8 PMA+Cal-I (out of 46, 17%) trajectories where the two-state diffusion model is preferred, Table 1. Thus, in all treatments we detected evidence of switching within trajectories with a similar level of preference. However, a proportion of the trajectories that preferred the two-state diffusion model showed extremely fast switching; we define fast switching as either ${\hat{p}}_{01}>0.1$ or ${\hat{p}}_{10}>0.1$, giving counts: DMSO, 3 trajectories; Cyto D, 5 trajectories; PMA, 5 trajectories; PMA+Cal-I, 2 trajectories, Table 1. Thus, over all treatments, for trajectories where the two-state diffusion model was preferred, we saw fast switching in 33% of trajectories.

**Table 1:** Model selection and proportion of time spent in the immobile state.

**Treatment**	DMSO	Cyto D	PMA	PMA+Cal-I
**Number of trajectories**	75	36	39	46
**Two-state model preferred** ^1^	16/67 (24%)	8/30 (27%)	13/36 (36%)	8/44 (18%)
**Two-state model preferred, fast switchers removed** ^1^ ^2^	13/64 (20%)	3/25 (12%)	8/31 (26%)	6/42 (14%)
***D*_1_ in immobile state** ^3^ **(two-state model preferred** ^1^ ^2^ **)**	0/13 (0%)	0/3 (0%)	0/8 (0%)	1/6 (17%)
***D* in immobile state** ^3^ **(one-state model preferred** ^1^ **)**	3/51 (6%)	4/22 (18%)	7/23 (30%)	19/36 (53%)
**Proportion of time in immobile state** ^4^	0.05	0.16	0.23	0.47
**Mean** ^5^ *D*_0_ ×10^4^ **nm^2^/s**	9.2 ± 1.8	9.4 ± 0.6	8.9 ± 0.8	7.0 ± 1.5
**Mean** ^5^ *D*_1_ ×10^4^ **nm^2^/s**	3.9 ± 0.7	4.9 ± 0.5	5.0 ± 0.6	2.5 ± 1.2
**Mean** ^6^ *D* ×10^4^ **nm^2^/s**	5.2 ± 0.4	5.5 ± 0.8	6.1 ± 0.9	5.1 ± 0.8
**Mean** ^7^ *D*_*immobile*_ ×10^4^ **nm^2^/s**	0.079 ± 0.025	0.057 ± 0.015	0.041 ± 0.007	0.062 ± 0.005

Diffusion coefficient units are nm^2^ s^−1^ with standard error based on the number of trajectories.

^1^ Model selection between approximate one-state and two-state diffusion models with measurement noise, see Methods, with trajectories with no strong model preference (−3 < log_*e*_ *B*_1*D*, 2*D*_ < 3) removed.

^2^ Fast switching trajectories (${\hat{p}}_{01}>0.1$ or ${\hat{p}}_{10}>0.1$) also removed.

^3^ Defined as log_*e*_ *D* < 8 or log_*e*_ *D*_1_ < 8, for mean posterior parameters *D*, *D*_1_ nm^2^ s^−1^ from one-state and two-state diffusion models with measurement noise.

^4^ Over all trajectories with either one-state or two-state model preference, with fast switching trajectories removed (i.e. Table notes 1 and 2 apply). For one-state preference, the proportion in the immobile state is 0 if log_*e*_ *D* > 8, 1 if log_*e*_ *D* > 8. For two-state preference, the proportion is 0 if log_*e*_ *D*_1_ > 8, and if log_*e*_ *D*_1_ < 8 the proportion of time that was spent in the *z* = 1 state (diffusion with *D* = *D*_1_), i.e. $1/N\;\sum_{i=1}^{N}\pi \left(z_{i}|X\right)$.

^5^ Over all posterior samples, for trajectories with two-state model preference, with fast switching trajectories removed.

^6^ Over all posterior samples, for trajectories with one-state model preference, restricted to log_*e*_ *D* > 8.

^7^ Over all posterior samples, for trajectories with one-state model preference, restricted to log_*e*_ *D* < 8.

This fast switching was similar to that observed for the fixed beads, questioning whether it is an experiment artifact or a true phenomena. We limited our analysis in the following to the slow (clear) switching trajectories and non-switching trajectories since the fast switchers clearly represent a different category of behaviour, irrespective of cause. Discounting those trajectories which have no strong model preference or are fast switchers, the proportion of trajectories where the two diffusion model was preferred over the one-state were: DMSO 13/64 (20%), Cyto D 3/25 (12%), PMA 8/31 (26%), PMA+Cal-I 6/42 (14%), Table 1.

**Fig 5:**
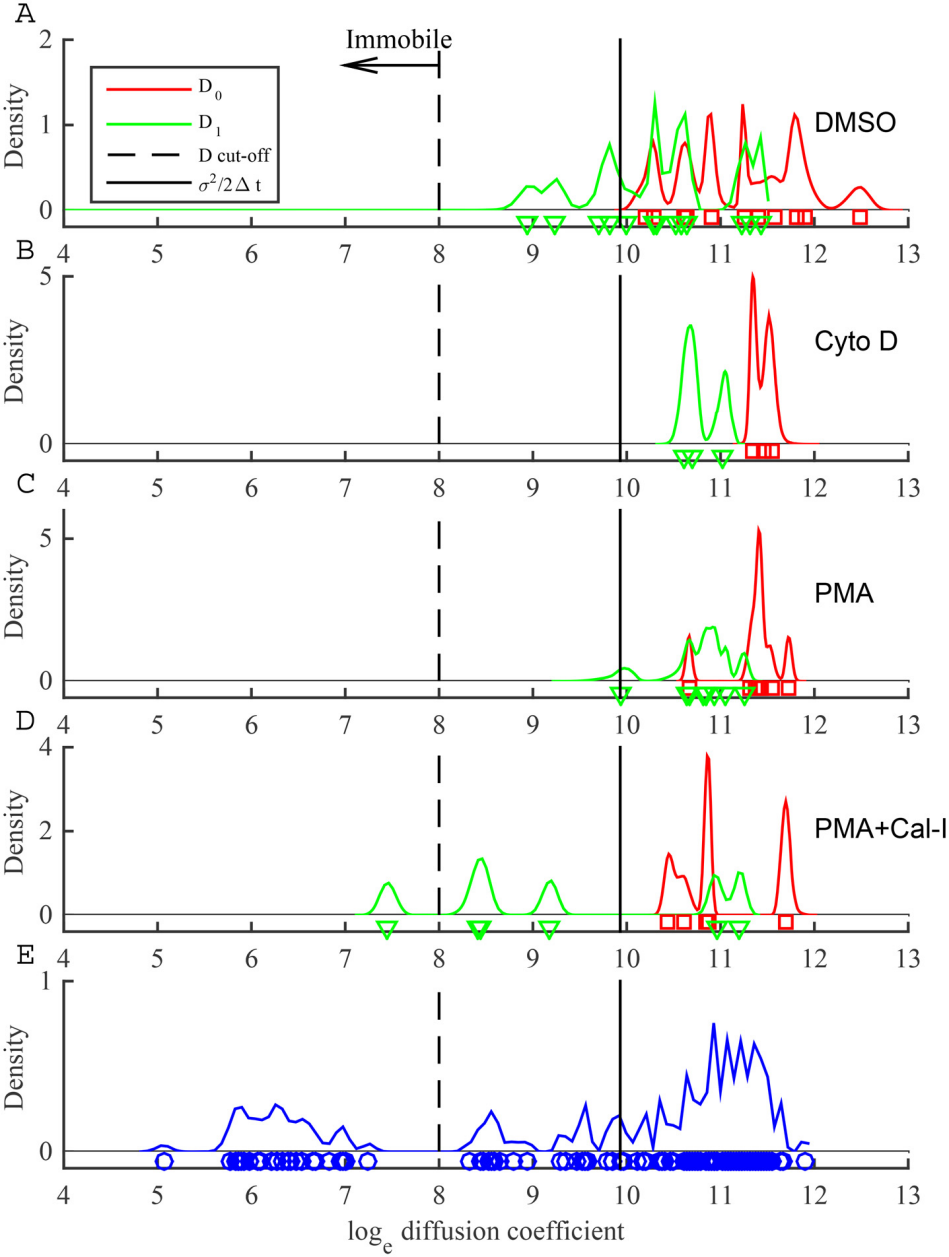
Posterior estimates of diffusion coefficients for single LFA-1 trajectories. (A-D) Pooled posterior samples of log_*e*_ *D*_0_ and log_*e*_ *D*_1_ for trajectories preferring the two-state diffusion model (fast switching, ${\hat{p}}_{01}>0.1$ or ${\hat{p}}_{10}>0.1$, trajectories removed). The posterior means for log_*e*_ *D*_0_ (red squares) and log_*e*_ *D*_1_ (green triangles), are also shown. Black line indicates value of *σ* ^2^/2Δ*t*. Dashed line indicates threshold used to categorise immobile and mobile diffusion states. Treatments: (A) DMSO, two-state model preferred for 13 trajectories; (B) Cyto D, 3 trajectories; (C) PMA, 8 trajectories; (D) PMA+Cal-I, 6 trajectories. (E) Pooled log_*e*_ *D* estimates and posterior means (blue circles) over all treatments, for trajectories where one-state diffusion model was preferred (132 trajectories).

**Fig 6:**
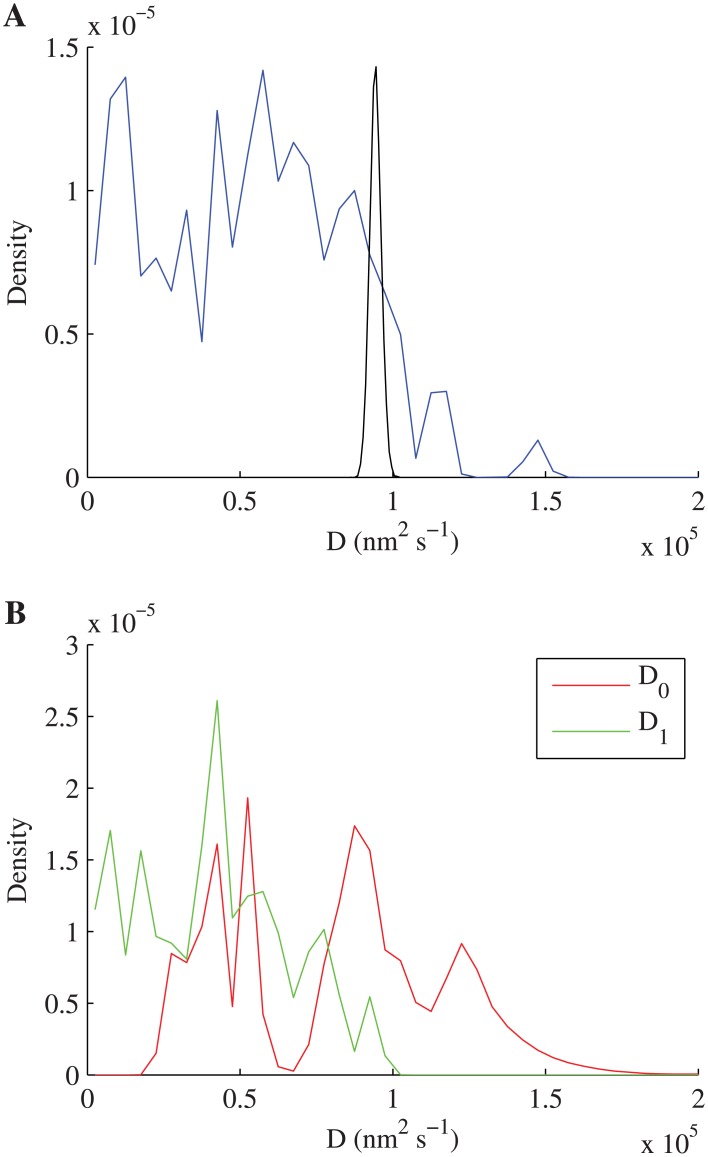
Pooled posterior distribution of diffusion coefficients for single LFA-1 trajectories. (A) Pooled posterior samples of *D* for trajectories where one-state diffusion model was preferred, restricted to log_*e*_ *D* > 8 (99 trajectories). The posterior distribution from a single trajectory (black line, DMSO treatment) is also plotted, normalised to equal height. (B) Pooled posterior samples of *D*_0_ and *D*_1_ for trajectories where two-state diffusion model was preferred, restricted to log_*e*_ *D*_1_ > 8 (29 trajectories), with fast switching (${\hat{p}}_{01}>0.1$ or ${\hat{p}}_{10}>0.1$) trajectories removed. One data point with *D*_0_ > 2 × 10^5^ nm^2^ s^−1^ not shown.

We next analysed the consistency of the diffusion coefficient estimates between trajectories. We note that the diffusion coefficients can be estimated below the measurement noise effective diffusion coefficient of *σ* ^2^/(2Δ*t*) since estimates are based on multiple time points, the error falling as *σ* ^2^/(2*n*Δ*t*) for *n* displacements. On the 4000 time points this gives a lower threshold of log(*D*) = 1.64, so well below the lowest inferred diffusion coefficient. For both sets of trajectories, those that preferred the one-state diffusion (*D*) or two-state diffusion (*D*_0_, *D*_1_), we computed the posterior mean diffusion coefficient and pooled their posterior distributions (in the full likelihood model, Figs 5 and 6). All four conditions demonstrated similar features:

- There are two distinct clusters in the *D* estimates (trajectories conforming to a single homogeneous diffusion): a high (mean) diffusion coefficient greater than 3.0 × 10^3^ nm^2^ s^−1^, and an essentially immobile state with a (mean) diffusion coefficient less than 3.0 × 10^3^ nm^2^ s^−1^, Fig 5E. Over the four conditions this split is very consistent, (S4 Fig). We refer to these as the mobile state, with *D* > 3.0 × 10^3^ nm^2^ s^−1^ (log_*e*_(*D*) > 8), and the immobile state, with *D* < 3.0 × 10^3^ nm^2^ s^−1^ (log_*e*_(*D*) < 8) (classified on the posterior mean of the diffusion coefficient). The mobile state may further decompose into a ‘low’ and ‘high’ diffusing state as the pooled *D* distribution is bimodal with separation at 2 × 10^4^ nm^2^/s, (Fig 6). The pooled distribution for *D*_0_ also suggests a mixed distribution, although the small number of trajectories (29) makes it difficult to reliably interpret.
- Trajectories with switching of the diffusion coefficient typically involve switching between two different mobile states; only 1 trajectory (out of 31) is observed to exhibit switching with the immobile state (Fig 5A–5D).
- The variance in the diffusion coefficient estimate for each trajectory is smaller than the variance between trajectories, (Fig 6); this implies that the bimodality (‘low’, ‘high’ diffusion coefficients) is further subdivided. This explains the distinct peaks in the pooled posterior distributions, Fig 5. Thus, there is variability in the diffusion coefficient estimates suggesting the presence of a heterogeneity amongst individual trajectories.

There are however differences between the four conditions. Most notably, the proportion of time in the immobile state is highest in PMA+Cal-I (47%, Table 1). This is significantly higher than DMSO (5%, *p* = 2.8 × 10^−8^), Cyto D (17%, *p* = 0.0091), and PMA (23% *p* = 0.031).

**Fig 7:**
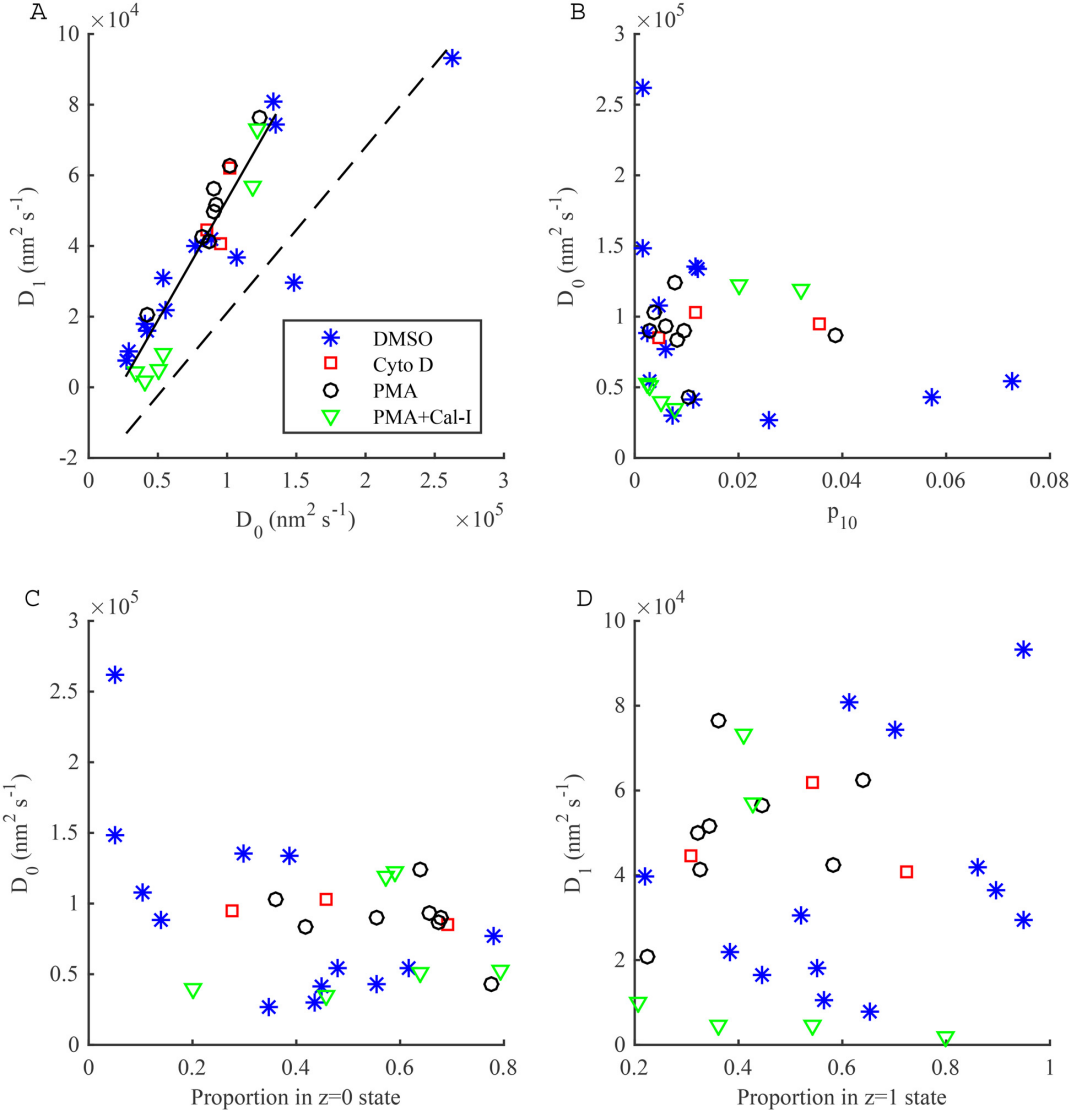
Dependences of parameter estimates from two-state diffusion model. (A-D) Scatter plots of posterior means for the two-state model with measurement noise, for trajectories where the approximate two-state diffusion model was preferred (fast switching, ${\hat{p}}_{01}>0.1$ or ${\hat{p}}_{10}>0.1$, trajectories removed). Treatments: DMSO, blue asterisks; Cyto D, red crosses; PMA, black circles; PMA+Cal-I, green triangles. In panel (A) the black solid line is a linear fit with two outlier trajectories removed, *D*_1_ = *aD*_0_ + *b*, *a* = 0.68, *b* = −1.5 × 10^4^ nm^2^ s^−1^; black dashed line is the double iterate, *D*_1_ = *a*(*aD*_0_ + *b*) + *b*.

For trajectories where the two-state diffusion model was preferred, (excluding the fast-switching trajectories), we examined if the diffusion coefficients between the two diffusive states are related (Fig 7A). The correlation coefficient is high (r = 0.84), whilst a linear relation is strongly suggested, *D*_1_ = 0.68*D*_0_ − 1.5 × 10^4^ nm^2^s^−1^, independent of treatment, using all points except the 2 outliers. This suggests that the switching events we are detecting are likely due to a single process. We also examined the relationship between *D*_0_ and *p*_10_, *D*_0_ and the time in the high (*z* = 0) diffusion state and *D*_1_ and the time in the low (*z* = 1) diffusion state, but found no correlation, Fig 7B–7D.

**Fig 8:**
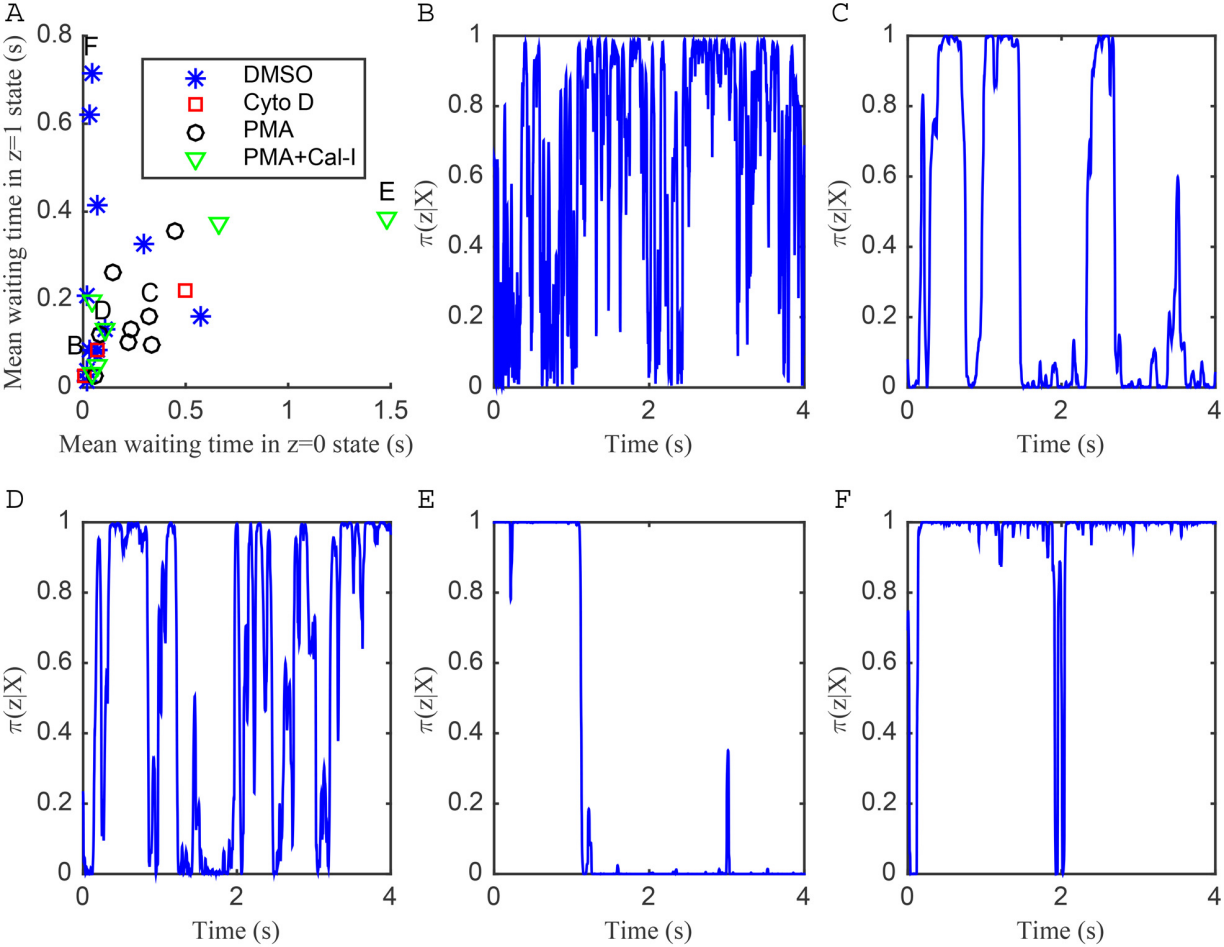
Mean waiting times and example trajectories showing confinement for two-state diffusion model fit to LFA-1 trajectories. (A) Mean waiting time in seconds ($1/(1000{\hat{p}}_{01})$ for *z* = 0 state, $1/(1000{\hat{p}}_{10}$) for *z* = 1 state) for trajectories where approximate two-state diffusion model was preferred (fast switching, ${\hat{p}}_{01}>0.1$ or ${\hat{p}}_{10}>0.1$, trajectories removed). Treatments: DMSO, blue asterisks; Cyto D, red squares; PMA black circles; PMA+Cal-I, green triangles. Labels B-F correspond to example confinement state trajectories in B-F. (B) DMSO treatment (mean waiting time in *z* = 0 state 0.02s, in *z* = 1 state 0.04s) (C) PMA treatment (*z* = 0 state 0.32s, *z* = 1 state 0.16s) (D) PMA treatment (*z* = 0 state 0.09s, *z* = 1 state 0.12s) (E) PMA+Cal-I treatment (*z* = 0 state 1.48s, *z* = 1 state 0.39s) (F) DMSO treatment (*z* = 0 state 0.04s, *z* = 1 state 0.72s).

We also examined the frequency of switching events for trajectories where the two-state diffusion model was preferred, excluding fast switching trajectories. Fig 8A plots the exponentially distributed waiting times in each state (i.e. the reciprocal of the inferred transition probabilities), demonstrating a broad range of values. Some trajectories exhibit fast transient switching (Fig 8A, trajectories clustered around origin, example in Fig 8B), although slower than that in stationary beads. Another group of trajectories switch less frequently, with the time in a single state on the order of tenths of seconds (Fig 8C and 8D). We also observe trajectories with very slow switching, Fig 8E is an example of a trajectory with a single switch point, whilst some trajectories spend the majority of time in the *z* = 0 (fast) state, with transient switching to the *z* = 1 (slow) state (Fig 8F). This variety suggests that multiple processes are affecting the waiting times since this range of behaviours would not be observed in a single exponential waiting time model.

**Fig 9:**
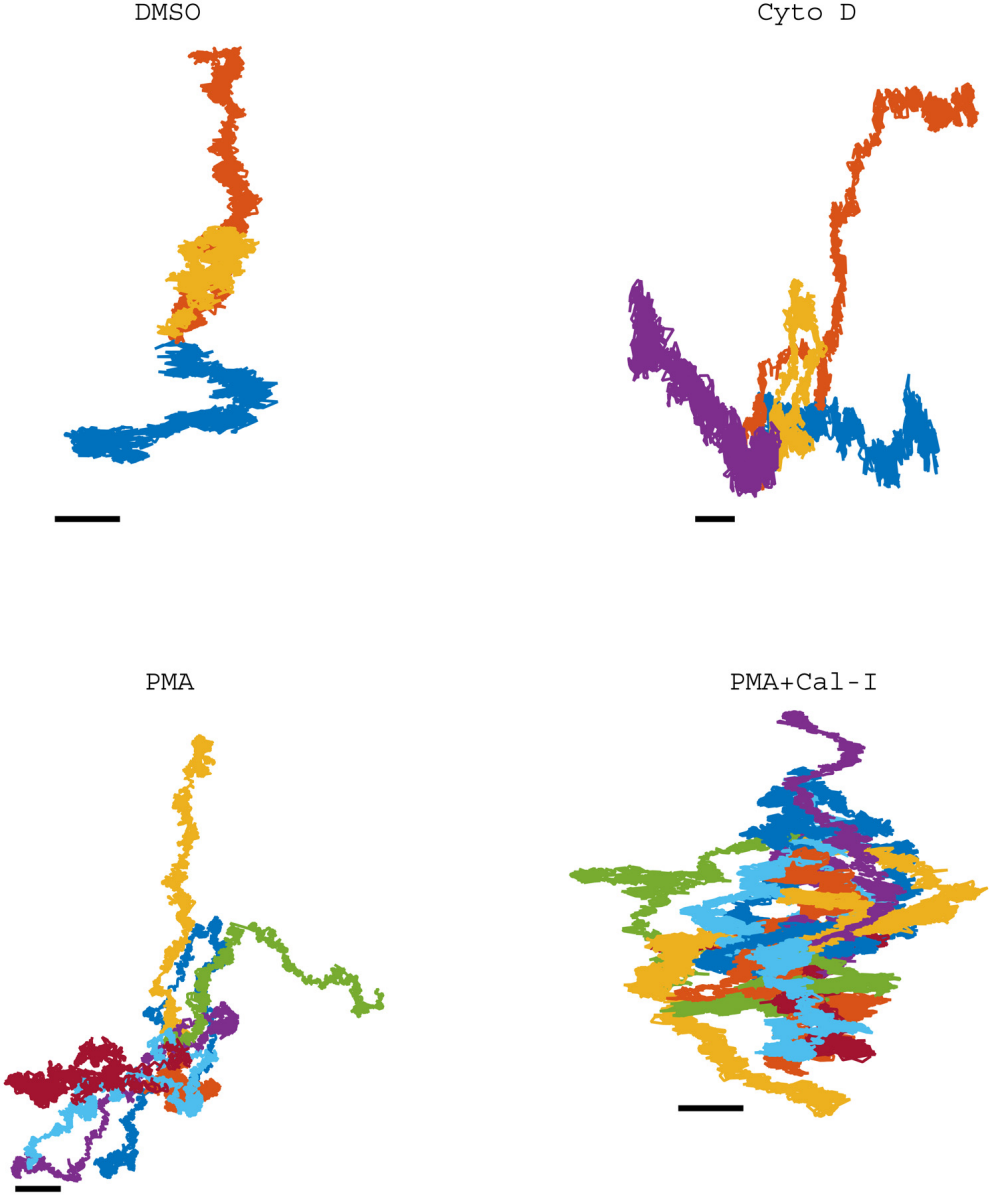
LFA-1 trajectories categorised as immobile (log_*e*_ *D* < 8 in the one-state model). Trajectories are from different cells, with the first timepoints shifted to (0, 0). Treatments: DMSO (3 of 75 in immobile state), Cyto D (4 of 36 in immobile state), PMA (7 of 39 in immobile state), PMA+Cal-I D (20 of 46 in immobile state). Scalebars: 50nm.

**Fig 10:**
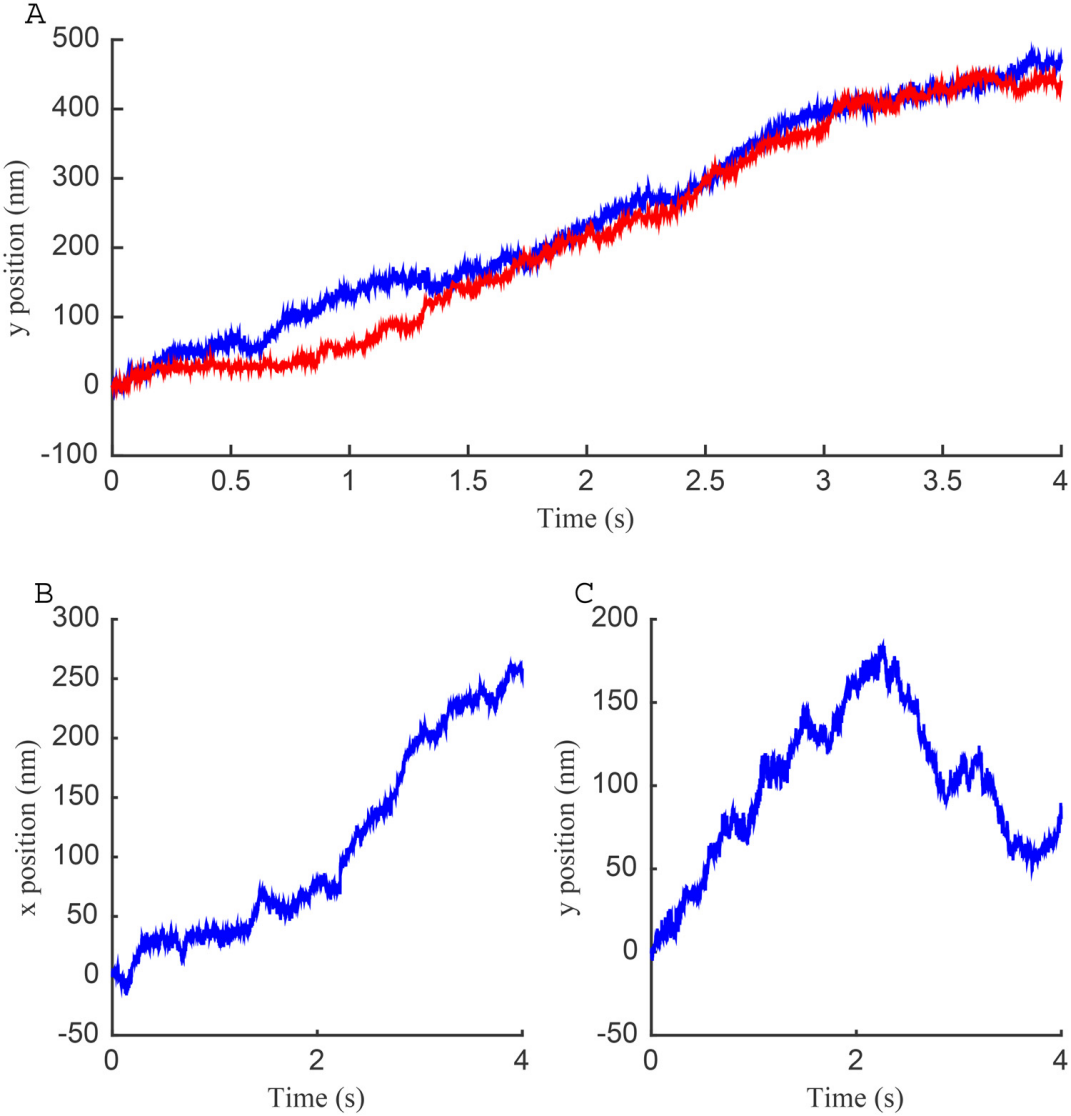
Linear drifts in LFA-1 trajectories categorised as immobile (log_*e*_ *D* < 8 in the one-state model). (A) Vertical displacements for two example trajectories. Blue line: DMSO treatment, ${\bar{v}}_{y}=117\;\text{nm}\;{\text{s}}^{-1}$; red line: PMA treatment, ${\bar{v}}_{y}=110\;\text{nm}\;{\text{s}}^{-1}$. (B-C) Displacements for a trajectory (PMA treatment) with a switch in drift direction. Estimated velocities: ${\bar{v}}_{x}=64\;\text{nm}\;{\text{s}}^{-1}$, ${\bar{v}}_{y}=80\;\text{nm}\;{\text{s}}^{-1}$, (average between 0 s and 2.25 s), ${\bar{v}}_{y}=-79\;\text{nm}\;{\text{s}}^{-1}$ (average between 2.25 s and 3.75 s), giving an average speed of 102 nm s^−1^.

We examined the trajectories identified to be in the immobile state in the one-state model. These trajectories show apparent phases of linear motion in arbitrary directions, Fig 9. Many trajectories have periods of consistent linear drifts in one direction, (examples in Fig 10), having speeds around 110 nm s^−1^. Some trajectories also have distinct changes in direction, (Fig 10B and 10C). Since the stationary beads do not show such drift, and the drift direction is variable, this is not due to microscope or sample drift (Jurkat cells in this assay being immobile [5]), but most likely reflects movements in the underlying actin cortex. These speeds are of the same order as the retrograde flow of actin in Jurkat cells (50 nm s^−1^) [50].

### Approximate versus exact models with measurement noise

**Fig 11:**
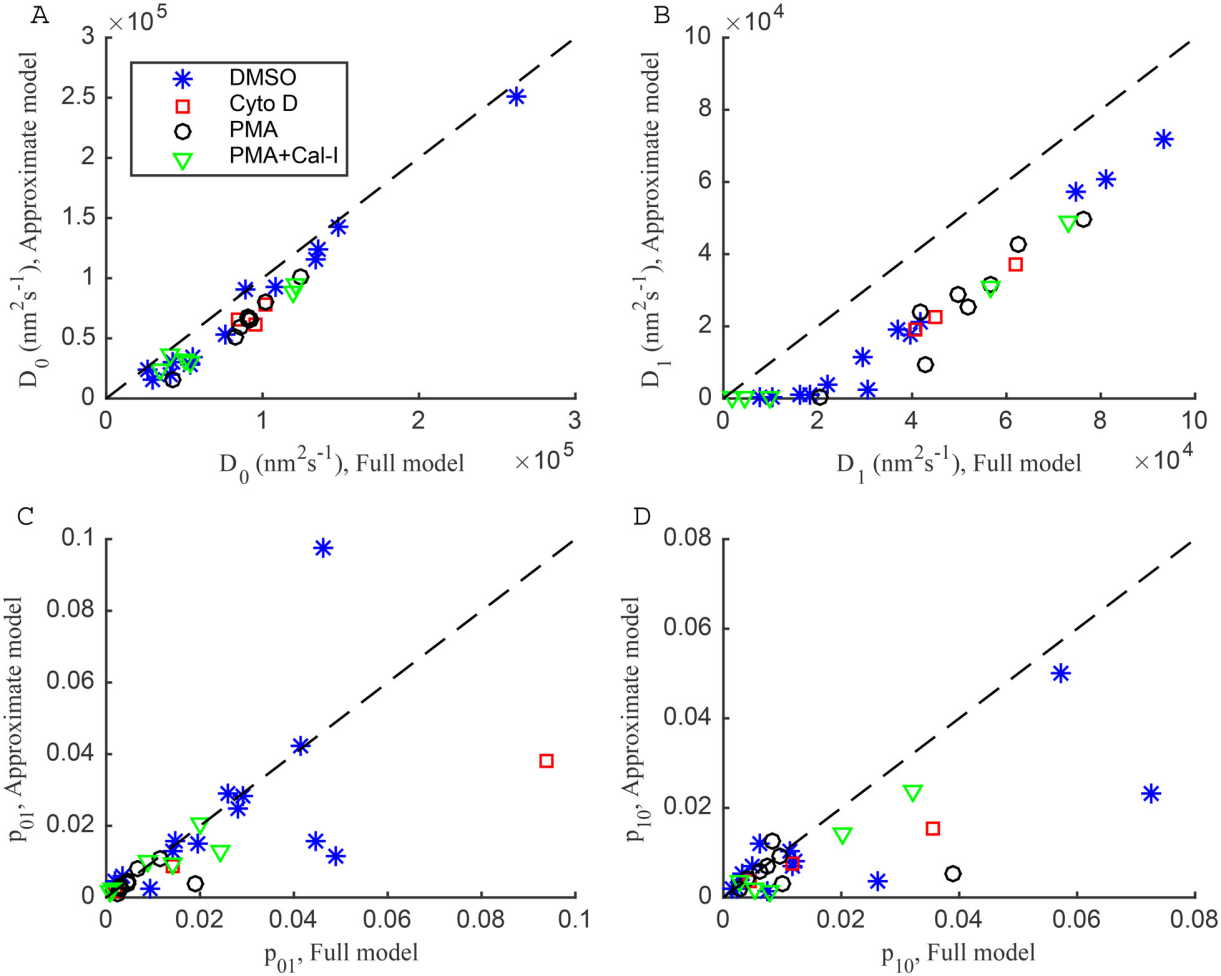
Comparison of parameter estimates for exact and approximate two-state diffusion models with measurement noise. (A-D) Scatter plots of two-state parameter estimates for exact model against approximate model, for 30 trajectories preferring the approximate two-state model (fast-switching, ${\hat{p}}_{01}>0.1$ or ${\hat{p}}_{10}>0.1$ in the exact model, trajectories removed). Line of equality is shown as dashed. Treatments: DMSO (blue asterisks), Cyto D (red squares), PMA (black circles), PMA+Cal-I (green triangles).

We used an approximate model (low noise limit) to compute the Bayes factor to determine which of the one and two-state diffusion models are preferred by each trajectory. This approximation is justified since it gives similar results to the (exact) model on individual trajectories (S5 Fig). On the LFA-1 trajectories that prefer the approximate model the hidden state correlation between these two algorithms is typically 80% or higher (S6 Fig). The diffusion coefficient estimates are also highly correlated (Fig 11), although they are lower under the approximation (significantly in a one-tailed Mann-Whitney test, with *p* = 0.02 for *D*_0_ and *p* = 0.001 for *D*_1_), indicating that failing to account for noise correlations in displacements introduces an estimation bias; this may potentially reduce the ability to detect two-state diffusion processes when the two diffusion coefficients are small (of order *σ* ^2^/Δ*t*). In fact we detect no intra-trajectory switchings with both diffusion coefficients below 2 × 10^4^ nm^2^s^−1^, Fig 5. However, trends are similar under both analyses—in common with the one-state and two-state diffusion models with measurement noise, we also see two clear subpopulations in the posterior mean and pooled posterior samples (S7 Fig), and a linear relationship between the *D*_0_ and *D*_1_ posterior means (S8 Fig). The approximate model therefore performs well on real data although it underestimates diffusion coefficients (Fig 11). Thus, we consider the approximate model sufficiently accurate for model selection SPT analysis, although parameter estimates are biased so we used the (exact) model for any estimates and interpretation after model selection.

## Discussion

We developed models and techniques for analysing single particle tracking data based on displacements between frames, including a Bayesian model selection methodology to ascertain whether the trajectory is more consistent with a one or two-state diffusion process. We confirmed the accuracy of our methods on simulated data. Two key elements of our analysis that distinguish it from other methods is the demonstration that model parameters can be estimated with high confidence from individual trajectories (1000 frames s^−1^ over 4 s), thereby not requiring trajectories to be pooled, and the inclusion of measurement noise in the trajectory inference, this propagating measurement error through to parameter estimates. We demonstrate that failure to do so leads to an inconsistency on stationary beads, (Fig 1), while use of the noiseless model on the LFA-1 trajectory data results in a doubling of the detection frequency of switching within trajectories (S2 Table). In part this is a consequence of the low signal to noise ratio in this data. An alternative method to deal with this low S/N is to subsample the data so that the signal is larger (S9 Fig). For example, modelling displacements over 4 time points reduces the effect of measurement noise. This unfortunately reduces the sample size so a balance is needed between increasing the S/N without losing too much data. We subsampled by applying a criteria per trajectory (see S2 Text and S10 Fig). This subsampling analysis gave comparative results to those obtained for the model with measurement noise on the whole data set, specifically similar numbers of trajectories showed preference for the two-state model while there is a high correlation in the model preference for each trajectory (S3 Table). This consistency between these two independent methods indicates that experimental or tracking artifacts are present, but effectively dealt with through these two alternative strategies.

Our methods were applied to single trajectories of LFA-1 tagged with latex beads under four conditions; this allowed us to show that a low but significant proportion of trajectories display within trajectory diffusion heterogeneity with switching between two distinct diffusion coefficients over a range of values (1.6 × 10^2^ − 2.6 × 10^5^ nm^2^ s^−1^), while the majority of trajectories conform to an homogeneous diffusion over the time scale of the trajectory. By treating each trajectory individually, rather than pooling trajectories in the analysis, we separate the heterogeneity due to this diffusive switching from a heterogeneity across trajectories, i.e. there are considerably more than two diffusive states. Previous LFA-1 studies that have pooled trajectory data miss a large component of this variability because pooling averages the heterogeneity. Basic trends are however consistent between the approaches, for instance Das *et al*., [32] demonstrated switching between two states which are comparable to our estimates (e.g. 8.5×10^4^ nm^2^s^−1^ and 3.1 ×10^4^ nm^2^s^−1^ for DMSO treatment). Three states of LFA-1 mobility have also been previously reported—“stationary”, “slow” and “fast” with estimated diffusion coefficients 1.4 ± 0.1 × 10^4^ nm^2^ s^−1^ and 5.6 ± 0.2 × 10^4^ nm^2^ s^−1^ for the slow and fast components respectively [36]. These are broadly in agreement with the two main peaks in the diffusion coefficient distribution (Fig 6A). However, our analysis demonstrates that fine detail of particle behaviour can be detected in single trajectories, in particular the diffusion coefficients can be estimated with high confidence thereby demonstrating that there is a large variability between the mobilities in individual trajectories (Fig 7). The interpretation of the distribution of observed (mobile) diffusion coefficients, (Figs 5E and 6) is subjective, for instance two Gaussians could be fitted to model the main peaks in Fig 6A, thereby splitting the mobile trajectories into what could be interpreted as slow and fast populations. However, as we demonstrate here the variability is not due to measurement noise, but is intrinsic to the tagged-LFA-1 molecules, our confidence intervals per trajectory being much smaller than the range. Thus, we prefer to interpret this as a graded diffusion coefficient in a continuum. We demonstrated that for LFA-1 there is switching between diffusion states on time scales of 10–100 ms, consistent with previous analyses, [21, 32]. The former demonstrated confinement within single trajectories, corresponding to our observation of the diffusion coefficient being reduced by a factor of 1.6–23.2 under switching (S11 Fig). However, our analysis extracts finer details than these two studies, specifically we show that there are multiple categories of behaviour, a low diffusion state consistent with immobility, and a sequence of higher diffusion states; the existence of more than two states was hinted at in the analysis of [32].

**Fig 12:**
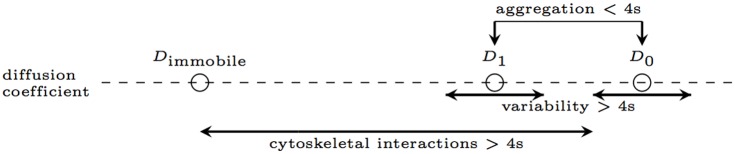
Observed variation in the diffusion coefficient of LFA-1 in single particle tracking trajectories, with proposed mechanisms.

The high variability of the estimated diffusion coefficients among both fast and slow trajectories may provide biological insight into the organisation of LFA-1 in the membrane. Clustering and cytoskeletal contacts are central to the regulation of LFA-1 in the membrane [51]. Previous work has found that the movement of clusters on live cells is dependent on the conformation of the receptor [5, 41]. We propose that the multi-state diffusion observed in the current analysis is a result of changes in the size of clusters, or the number of cytoskeletal contacts for those clusters. The relationship in Fig 7A suggests that the switching events we are detecting are all due to a common process. One interpretation is that we are observing diffusing aggregates of LFA-1, either in protein islands [52], or due to multiple attachments of LFA-1 molecules with the bead, a change in the aggregate size by 1 corresponding to a switch in the diffusion coefficient. We hypothesise that the diffusion coefficient reflects the size of the aggregate; the cross section of a receptor or complex in the membrane has a predictable effect on its diffusion [53]. However, the variability in the (high) diffusion coefficients that we observe is inconsistent with this process alone. Since diffusion coefficients are observed along the straight line in Fig 7A, there is an heterogeneity that determines the diffusion coefficient by smaller increments, and is presumably also responsible for the large variability in the switching frequency, (Fig 8). We thus have a hierarchy of processes: on time scales less than 4 s we observe changes in the aggregate size producing large changes in the diffusion coefficient according to Fig 7A, and these aggregates are also affected by a slower process that results in a finer heterogeneity (Figs 6 and 12). A potential mechanism is cytoskeletal attachment, with the number of attachments increasing with aggregate size thereby increasing the drag, and a sufficiently large number of these interactions making the receptor aggregate immobile, giving an interpretation of the non-zero intercept of the *D*_0_/*D*_1_ relationship in Fig 7. This is consistent with calpain inhibition having the highest level of immobility, Table 1, since calpain cleaves the talin head domain and releases LFA-1 from the cytoskeleton [42]. The fact that the mobile diffusion coefficient is reduced under calpain treatment, Table 1, also supports the fact that cytoskeletal interactions are contributing to the aggregate drag. We also demonstrated that the immobile states (detected predominantly as immobile throughout) typically have a slow linear drift, with speeds of around 110 nm s^−1^. We suggest that these correspond to LFA-1 (possibly clusters) strongly bound to the actin cortex, and these drift phases correspond to cortex remodelling under actin (de)polymerisation, myosin contraction or retrograde flux, [50]. Such drift was also detected by MSD analysis as super-diffusion (*α* > 1) [5].

Alternative interpretations are possible, we cannot discount the possible effect of the multivalent probe on the experiment. It is possible that changes in diffusive states are the result of different numbers of contacts between the probe and receptors in the membrane. Resolving these issues will require a larger amount of data, of the order of 100s of trajectories, and ideally across different sized and variable Ab density beads.

Our analysis thus highlights the importance of large trajectory databases, with trajectory resolution and length reflecting the dynamics of the system. Quantum dots are an attractive option, since they are smaller than typical labelling molecules, and provide long trajectories [26, 54]. Ideally data on different tagging regimes is also needed to distinguish tag artifacts from molecule dynamics. With such data, sophisticated (HMM) models of temporal heterogeneity can be utilised, extending for instance to multi-diffusion states, confinement zones and drift, implemented with the algorithms and techniques demonstrated here to analyse individual trajectories. These methods applied to large trajectory databases of long high-resolution trajectories will be an important contribution to the understanding of the complexity of membrane organisation and the multiple diffusion modalities present in cells [8].

## Supporting Information

S1 Text Supplementary mathematical derivations. Step by step calculation of likelihoods, marginal likelihoods, and MCMC algorithms, for one-state and two-state diffusion models described in the Methods section.
(PDF)

S2 Text Subsampling trajectories to reduce the effect of measurement noise. Justification and description of the subsampling approach described in the Discussion.
(PDF)

S1 Files Single particle tracking trajectories in MAT and HDF5 file formats. All trajectories are 1000 frames s^−1^. Treatments: DMSO, 75 trajectories of length 4 s; Cyto D, 36 trajectories of length 4 s; PMA, 39 trajectories of length 4 s; PMA+Cal-I, 46 trajectories of length 4 s; stationary beads, 3 trajectories of length 2 s.
(ZIP)

S1 Algorithms Pseudocode for algorithms. Pseudocode for one-state and two-state diffusion model MCMC algorithms.
(PDF)

S1 Table Model selection results for different Bayes factor thresholds. (PDF)

S2 Table Influence of noise and S/N on model selection preferences between one-state and two-state diffusion models on LFA-1 data (157 trajectories). (PDF)

S3 Table Comparison of model selection for approximate measurement noise models and subsampled trajectories. (PDF)

S1 Fig Fit of the exact two-state diffusion model with measurement noise to a simulated two-state diffusion trajectory. (A) The posteriors for the two diffusion coefficients with true *D*_1_ (red square) and *D*_0_ (blue asterisk) values plotted, (B) corresponding samples for *D*_0_ (red) and *D*_1_ (blue) including burn-in (dashed line). (C) Posteriors for the switching probabilities per frame, with true *p*_01_ (blue asterisk) and *p*_10_ (red square) values plotted (D) corresponding samples for *p*_01_ (red) and *p*_10_ (blue) including burn-in (dashed line). (E) Diffusion state inference (blue, dashed) and true state (red) shown as the probability of being in the low diffusion state. (F) Trajectory coloured by the probability of being in the low diffusion state. Colour scale represents *π*(**z** = 1∣**X**) from 0 (blue, high diffusion state) to 1 (green, low diffusion state). Colorbar length: 100nm. (G,H) Mean inferred position of **U** (blue, dashed) and true particle position (red). Simulated measurement noise and measurement noise for inference both set to *σ* ^2^ = 41.09nm. Data from 20000 MCMC steps with a 10000 step burn-in. See Methods for priors and initial conditions.
(EPS)

S2 Fig Diffusion coefficients separated by a factor of 1.5 can be detected on the exact two-state diffusion model with measurement noise. (A-D) The posteriors for the two diffusion coefficients with true *D*_1_ (red square) and *D*_0_ (blue asterisk) values plotted; true *D*_0_, *D*_1_ differ by a factor of 1.5 (A,C) and 2 (B,D), with low (A,B) and high (C,D) diffusion coefficients. (E-H) Corresponding diffusion state inference (blue, dashed) and true state (red) shown as the probability of being in the low diffusion state. The transition probabilities for all trajectories were *p*_01_ = 0.005, *p*_10_ = 0.005. Measurement noise set to *σ* ^2^ = 41.09nm for both the simulated data and inference algorithm. Trajectories comprise 4000 frames. Data from 20000 MCMC steps with a 10000 step burn-in. See Methods for priors and initial conditions.
(EPS)

S3 Fig Model selection between one-state and two-state diffusion models with measurement noise on simulated trajectories. Box and whisker plots of log Bayes factors for simulated datasets (trajectories are length 4 s with 1000 frames s^−1^). Trajectories with log Bayes factor outside 1.5 times IQR are plotted as outliers (red crosses). (A) Parameters for simulated data (50 trajectories for each model): two-state and two-state with noise, *D*_0_ = 10^5^ nm^2^s^−1^, *D*_1_ = 2 × 10^4^ nm^2^s^−1^, *p*_01_ = 0.01, *p*_10_ = 0.01; one-state and one-state with noise, *D* = 10^5^ nm^2^s^−1^. (B) Parameters for simulated data (20 trajectories for each model): two-state and two-state with noise, *D*_0_ = 5 × 10^5^ nm^2^s^−1^, *D*_1_ = 2 × 10^4^ nm^2^s^−1^, *p*_01_ = 0.01, *p*_10_ = 0.01; one-state and one-state with noise, *D* = 5 × 10^5^ nm^2^s^−1^. Measurement noise in the simulations was *σ* ^2^ = 41.09 nm. MCMC runs were 20000 steps with a 10000 step burn in, with measurement noise fixed as *σ* ^2^ = 41.09 nm.
(EPS)

S4 Fig Posterior estimates of diffusion coefficients for single LFA-1 trajectories. (A-D) Pooled posterior samples of log_*e*_ *D* for trajectories preferring the one-state diffusion model. The posterior means (blue circles) are also shown. Black line indicates value of *σ* ^2^/2Δ*t*. Treatments: (A) DMSO, one-state model preferred for 51 trajectories; (B) Cyto D, 22 trajectories; (C) PMA, 23 trajectories; (D) PMA+Cal-I, 36 trajectories.
(EPS)

S5 Fig Fit of the approximate two-state diffusion model with measurement noise to an LFA-1 trajectory (PMA+Cal-I treatment). Compare to Fig 3 fitting the exact noise model to the same trajectory. (A) The posteriors for the two diffusion coefficients, (B) corresponding samples (12 independent chains plotted in the same colour) for *D*_0_ and *D*_1_ including burn-in (dashed line), (C) posteriors for the switching probabilities per frame, (D) corresponding samples (12 chains) for *p*_01_ and *p*_10_ including burn-in (dashed line), (E) State inference shown as the probability of being in the low diffusion state, (F) trajectory coloured by the probability of being in the low diffusion state. Colour scale represents *π*(**z** = 1∣**X**) from 0 (blue, high diffusion state) to 1 (green, low diffusion state). Colorbar length: 100nm. Data from 12 parallel chains of 20000 MCMC steps with a 10000 step burn-in. Priors, see Methods.
(EPS)

S6 FigComparison of hidden state inference for the exact and approximate two-state diffusion models with measurement noise.(A) Correlation between inferred hidden state **z** for each model, pooled across all treatments for 30 trajectories preferring the approximate two-state model (fast-switching, ${\hat{p}}_{01}>0.1$ or ${\hat{p}}_{10}>0.1$ in the exact model, trajectories removed). (B) Example hidden state posterior for approximate two-state model (blue) and exact two-state model (red) for a single trajectory (PMA+Cal-I treatment).(EPS)

S7 FigPosterior estimates of diffusion coefficients from fitting approximate two-state diffusion model with measurement noise to LFA-1 trajectories.(A-D) Pooled posterior samples of log_*e*_ *D* (blue lines) for trajectories with one-state diffusion model preference, or log_*e*_ *D*_0_ (red lines) and log_*e*_ *D*_1_ (green lines) for trajectories with two-state diffusion model preference (fast switching, ${\hat{p}}_{01}>0.1$ or ${\hat{p}}_{10}>0.1$, trajectories removed). Also plotted are the posterior means of log_*e*_ *D* for each trajectory with one-state model preference (blue circles), and posterior means of log_*e*_ *D*_0_ (red squares) and log_*e*_ *D*_1_ (green triangles) for each trajectory with two-state model preference. Black line indicates value of *σ* ^2^/2Δ*t*. Treatments: (A) DMSO, one-state model preferred for 51 trajectories, two-state model preferred for 14 trajectories; (B) Cyto D, 22 one-state, 5 two-state; (C) PMA 23 one-state, 14 two-state (D) PMA+Cal-I, 36 one-state, 7 two-state. (E) Pooled log_*e*_ *D* estimates and posterior means for each trajectory over all treatments for trajectories where one-state diffusion model was preferred.(EPS)

S8 FigDependences of parameter estimates from approximate two-state diffusion model with measurement noise.(A-D) Scatter plots of posterior means of stated parameters for approximate two-state model with measurement noise inference, for trajectories where the approximate two-state diffusion model was preferred, (fast switching, ${\hat{p}}_{01}>0.1$ or ${\hat{p}}_{10}>0.1$, trajectories removed). Treatments: DMSO, blue asterisks; Cyto D, red squares; PMA black circles; PMA+Cal-I, green triangles. In panel (A) the black solid line is a linear fit with two outlier trajectories removed, *D*_1_ = *aD*_0_ + *b*, *a* = 0.57, *b* = −1.3 × 10^4^ nm^2^ s^−1^; black dashed line is double iterate, *D*_1_ = *a*(*aD*_0_ + *b*) + *b*.(EPS)

S9 FigSignal to noise against subsampling rate for LFA-1 trajectories.The “signal” is the average variance of individual displacements of LFA-1 trajectories over all treatments, and the “noise” is the average variance of individual displacements for three stationary latex bead trajectories. Trajectories were subsampled at rate *n* by including only every *n*th timepoint, giving a trajectory of length $\left\lfloor \frac{N}{n}\right\rfloor$.(EPS)

S10 Fig Mean square displacement plots for three SPT trajectories. Red line is the straight line fit to the optimum number of MSD points to use when estimating the diffusion coefficient *D* [15]. (A) Stationary latex bead. (B) Slow diffusing LFA-1 trajectory (PMA+Cal-I treatment). (C) Fast diffusing LFA-1 trajectory (PMA+Cal-I treatment).
(EPS)

S11 FigPosterior estimates of *D*_0_/*D*_1_ ratio for the two-state diffusion model with measurement noise fitted to LFA-1 trajectories.Posterior mean *D*_0_/*D*_1_, for trajectories where two-state diffusion model was preferred, pooled across treatments (fast switching, ${\hat{p}}_{01}>0.1$ or ${\hat{p}}_{10}>0.1$, trajectories removed).(EPS)
