# Supplementary material for: Detection of Diffusion Heterogeneity in Single Particle Tracking Trajectories Using a Hidden Markov Model with Measurement Noise Propagation
Source: PLoS One. 2015 Oct 16;10(10):e0140759. doi: 10.1371/journal.pone.0140759 (PMC4608688; doi:10.1371/journal.pone.0140759)
Supplement: S1 Text — Step by step calculation of likelihoods, marginal likelihoods, and MCMC algorithms, for one-state and two-state diffusion models described in the Methods section. (PDF) [file pone.0140759.s001.pdf]

S1 Text: Detection of Diffusion Heterogeneity in Single Particle Tracking Trajectories using a Hidden Markov Model with Measurement Noise Propagation

This document includes step by step calculation of likelihoods, marginal likelihoods and MCMC algorithms, for one-state and two-state diffusion models as described in the Methods section.

One-state diffusion model marginal likelihood calculation

The marginal likelihood is defined

$$\pi(\mathbf{X}|M_{1D}) = \int_0^\infty \mathrm{d}D\, \pi(\mathbf{X}|D, M_{1D})\pi(D)$$

since $\pi(D) = \text{Uniform}(0, D_{max})$ we can write

$$\pi(\mathbf{X}|M_{1D}) = \frac{1}{D_{max}} \int_0^{D_{max}} \mathrm{d}D\, \pi(\mathbf{X}|D, M_{1D})$$

changing variables from D to D^{-1} gives

$$\begin{aligned}
\pi(\mathbf{X}|M_{1D}) &= \frac{1}{D_{max}} \int_{1/D_{max}}^\infty \mathrm{d}D^{-1}\, D^2 \pi(\mathbf{X}|D, M_{1D}) \\
&= \frac{1}{D_{max}} \int_{1/D_{max}}^\infty \mathrm{d}D^{-1}\, D^2 \prod_{i=1}^N \frac{1}{4\pi D \Delta t_i} \exp\left(-\sum_{i=1}^N \frac{\Delta X_i^2}{4D\Delta t_i}\right) \\
&= \frac{1}{D_{max}} \prod_{i=1}^N \frac{1}{4\pi\Delta t_i} \int_{1/D_{max}}^\infty \mathrm{d}D^{-1} \left(\frac{1}{D}\right)^{N-2} \exp\left(-\frac{1}{D}\sum_{i=1}^N \frac{\Delta X_i^2}{4\Delta t_i}\right).
\end{aligned}$$

In general $\int_x^\infty \mathrm{d}t\, t^{\alpha-1}\mathrm{e}^{-\beta t} = \frac{1}{\beta^\alpha}\Gamma(\alpha, \beta x)$, where $\Gamma(\alpha, \beta x)$ is an upper incomplete gamma function. Using this gives

$$\pi(\mathbf{X}|M_{1D}) = \frac{1}{D_{max}} \prod_{i=1}^N \frac{1}{4\pi\Delta t_i} \left(\sum_{i=1}^N \frac{\Delta X_i^2}{4\Delta t_i}\right)^{1-N} \Gamma\left(N-1, \frac{1}{D_{max}} \sum_{i=1}^N \frac{\Delta X_i^2}{4\Delta t_i}\right).$$

Approximate one-state diffusion model with measurement noise

We consider a trajectory $\mathbf{X}$ subject to Gaussian observation error, with fixed localisation accuracy σ^2. By a result in [1] (also see the section Approximation to the likelihood for one-state diffusion model with measurement noise) an approximation for the likelihood of $\mathbf{X}$ given D is

$$\pi(\mathbf{X}|D) = \prod_{i=1}^N N\left(\Delta X_i; 0, 2(D\Delta t_i + \sigma^2)\right).$$

The associated posterior is

$$\pi(D|\mathbf{X}) \propto \pi(D) \prod_{i=1}^{N} N\left(\Delta X_i; 0, 2D\Delta t_i + 2\sigma^2\right),$$

which can be sampled using a Metropolis-Hastings algorithm. We set $\pi(D) = \text{Unif}(D; 0, D_{max})$, and use a random walk sampler (RW MCMC) with a symmetric Gaussian proposal, $q(D \to D') = N(D'; D, S_D)$, giving the acceptance probability

$$\alpha(D \to D') = \min\left\{1, \frac{\prod_{i=1}^{N} N\left(\Delta X_i; 0, 2(D'\Delta t_i + \sigma^2)\right)}{\prod_{i=1}^{N} N\left(\Delta X_i; 0, 2(D\Delta t_i + \sigma^2)\right)}\right\} \mathbb{1}_{[0,D_{max}]}(D).$$

Thus, any moves outside $[0, D_{max}]$ are automatically rejected. The value of S_D is tuned during the burn-in to ensure that the acceptance rate is approximately 0.25 [2]. The MCMC sampler is also given as pseudocode in S1 Algorithms.

Approximate two-state diffusion model with measurement noise

We now add fixed localisation error to the previous two-state diffusion hidden Markov model. Using the same approximation to the likelihood as the approximate one-state model we can write

$$\begin{aligned} z_i|_{z_{i-1}} &\sim \text{Bernoulli}(z_{i-1}(1-p_{10}) + (1-z_{i-1})p_{01}) \\ \Delta X_i|_{z_i} &\sim N(0, 2(D_{z_i}\Delta t_i + \sigma^2)). \end{aligned}$$

Letting $\theta = \{D_0, D_1, p_{01}, p_{10}\}$ we can write the posterior as

$$\begin{aligned} \pi(\theta, \mathbf{z}|\mathbf{X}) \propto \pi(\theta)\pi(z_1|\theta) &\prod_{i=1}^{N} N(\Delta X_i; 0, 2(D_{z_i}\Delta t_i + \sigma^2)) \\ \times &\prod_{i=1}^{N-1} \text{Bernoulli}(z_{i+1}; z_i(1-p_{10}) + (1-z_i)p_{01}). \end{aligned} \tag{1}$$

We use the same priors on D_0, D_1, p_{01}, p_{10} and z_1 as in the two-state diffusion model without measurement noise, given in equation (7), main text. D_0, D_1 are updated with Metropolis-Hastings moves. The proposals are Gaussians centred at the current value $q(D_0 \to D_0') = N(D_0'; D_0, S_{D_0}), q(D_1 \to D_1') = N(D_1'; D_1, S_{D_1})$ and the acceptance probabilities are

$$\begin{aligned} \alpha(D_0 \to D_0'|\mathbf{z}, \mathbf{X}) &= \min\left\{1, \frac{\prod_{z_i=0} N(\Delta X_i; 0, 2(D_0'\Delta t_i + \sigma^2))}{\prod_{z_i=0} N(\Delta X_i; 0, 2(D_0\Delta t_i + \sigma^2))}\right\} \mathbb{1}_{[0,D_{max}]}(D_0') \\ \alpha(D_1 \to D_1'|\mathbf{z}, \mathbf{X}) &= \min\left\{1, \frac{\prod_{z_i=1} N(\Delta X_i; 0, 2(D_1'\Delta t_i + \sigma^2))}{\prod_{z_i=1} N(\Delta X_i; 0, 2(D_1\Delta t_i + \sigma^2))}\right\} \mathbb{1}_{[0,D_{max}]}(D_1'). \end{aligned}$$

S_{D_0}, S_{D_1} are tuned during the burn-in to ensure an acceptance rate of approximately 0.25. We also impose the condition that $D_0 < D_1$, which we enforce after the MCMC run as follows: if the posterior means $\hat{D}_0 > \hat{D}_1$ then we swap the D_0, D_1 chains, swap the p_{01}, p_{10} chains, and swap the 0 and 1 states in the hidden state $\mathbf{z}$ throughout the run. This is possible because although state identity switching $(0 \leftrightarrow 1)$ is possible because of a permutation symmetry during a run, it isn't observed to occur. The updates for the transition probabilities are Gibbs moves, identical to the two-state model without measurement noise, given by equations (15) and (16), main text. The $\mathbf{z}$ update is similar to the other two-state models, the conditional is

$$\begin{aligned} \pi(z_i|z_{i-1}, z_{i+1}, ..) \propto\ &\text{Bernoulli}(z_i; z_{i-1}(1-p_{10}) + (1-z_{i-1})p_{01}) \\ &\times N(\Delta X_i; 0, 2D_{z_i}\Delta t_i + 2\sigma^2) \\ &\times \text{Bernoulli}(z_{i+1}; z_i(1-p_{10}) + (1-z_i)p_{01}). \end{aligned} \tag{2}$$

And again the update is

$$z_i\Big|_{\theta,\mathbf{U},z_{i\pm1}} \sim \text{Bernoulli}\left(\pi(z_i = 1|z_{i-1}, z_{i+1}, \theta, \mathbf{X})\right).$$

At the endpoints $i = 1$ and $i = N$ we have

$$\pi(z_1|z_2, \theta, \mathbf{X}) \propto N(\Delta X_1; 0, 2D_{z_1}\Delta t_1 + 2\sigma^2)\text{Bernoulli}\left(z_2; z_1(1 - p_{10}) + (1 - z_1)p_{01}\right) \quad (3)$$

$$\pi(z_N|z_{N-1}, \theta, \mathbf{X}) \propto \text{Bernoulli}\left(z_N; z_{N-1}(1 - p_{10}) + (1 - z_{N-1})p_{01}\right) N(\Delta X_N; 0, 2D_{z_N}\Delta t_N + 2\sigma^2). \quad (4)$$

Pseudocode for this MCMC sampler is given in S1 Algorithms.

Approximation to the likelihood for one-state diffusion model with measurement noise

(This method is mentioned in reference [1].) Consider a 2D trajectory observed with experimental noise with known localisation accuracy σ^2. Let $\{U_i\}_{i=1}^{N+1}$ be the underlying particle position and $\{X_i\}_{i=1}^{N+1}$ be the observed positions. For each time step

$$U_i - U_{i-1} \sim N(0, 2D\Delta t_{i-1})$$
$$X_i \sim N(U_i, \sigma^2).$$

Which we can write as (summing two Gaussians)

$$X_i - X_{i-1} \sim N(U_i - U_{i-1}, 2\sigma^2)$$

shifting the mean

$$X_i - X_{i-1} \sim N(0, 2\sigma^2) + U_i - U_{i-1}$$

since $U_i - U_{i-1} \sim N(0, 2D\Delta t_{i-1})$ we can write

$$X_i - X_{i-1} \sim N(0, 2D\Delta t_{i-1} + 2\sigma^2).$$

So we know that the measured displacement then satisfies $X_{i+1}|X_i \sim X_i + N(0, 2D\Delta t_i + 2\sigma^2)$, which suggests that the likelihood is given by

$$\pi(\mathbf{X}|D) = \prod_{i=1}^{N} N(\Delta X_i; 0, 2D\Delta t_i + 2\sigma^2). \quad (5)$$

However, this is only true if the displacements are independent, which not the case since the displacements $U_{i+1} - U_i$ and $U_i - U_{i-1}$ both depend on the measurement noise $U_i - X_i$ at time point i. However, we demonstrate that equation (5) is sufficient for model selection, see Results.

Log likelihood for approximate two-state diffusion model with measurement noise

We use a modified version of the Das *et al.* forward algorithm [3] to calculate $\pi(\mathbf{X}|\theta)$. The initial forward probabilities in log scale are

$$\log_e \alpha_1(z_1 = 0) = \log_e \frac{p_{10}}{p_{10} + p_{01}} + \log_e \pi(\Delta X_1|z_1 = 0, D_0, D_1)$$
$$\log_e \alpha_1(z_1 = 1) = \log_e \frac{p_{01}}{p_{10} + p_{01}} + \log_e \pi(\Delta X_1|z_1 = 1, D_0, D_1)$$

where $\log_e \pi(\Delta X_i | z_i, D_0, D_1) = N(\Delta X_i; 0, 2(D_{z_i} \Delta t_i + \sigma^2))$ for $i = 1..N$. The recursion for $i = 2$ to $i = N$ is then

$$\log_e \alpha_i(z_i = 0) = \log_e \Big[e^{\log_e \alpha_{i-1}(z_{i-1}=0) + \log_e(1-p_{01}) + \log_e \pi(\Delta X_i | z_i=0, D_0, D_1)} + e^{\log_e \alpha_{i-1}(z_{i-1}=1) + \log_e(p_{10}) + \log_e \pi(\Delta X_i | z_i=0, D_0, D_1)} \Big]$$

$$\log_e \alpha_i(z_i = 1) = \log_e \Big[e^{\log_e \alpha_{i-1}(z_{i-1}=1) + \log_e(p_{01}) + \log_e \pi(\Delta X_i | z_i=1, D_0, D_1)} + e^{\log_e \alpha_{i-1}(z_{i-1}=0) + \log_e(1-p_{10}) + \log_e \pi(\Delta X_i | z_i=1, D_0, D_1)} \Big].$$

And the final likelihood is

$$\pi(\mathbf{X}|\theta) = \log_e \left[e^{\log_e \alpha_N(z_N=0)} + e^{\log_e \alpha_N(z_N=1)} \right].$$

References

[1] Michalet X. Mean square displacement analysis of single-particle trajectories with localization error: Brownian motion in an isotropic medium. Physical review E, Statistical, nonlinear, and soft matter physics. 2010 Oct;82(4 Pt 1):041914.

[2] Roberts GO, Gelman A, Gilks WR. Weak Convergence And Optimal Scaling Of Random Walk Metropolis Algorithms. The Annals of Applied Probability. 1997;7(1):110–120.

[3] Das R, Cairo CW, Coombs D. A hidden Markov model for single particle tracks quantifies dynamic interactions between LFA-1 and the actin cytoskeleton. PLoS computational biology. 2009 Nov;5(11):e1000556.
