# Supplementary material for: Detection of Diffusion Heterogeneity in Single Particle Tracking Trajectories Using a Hidden Markov Model with Measurement Noise Propagation
Source: PLoS One. 2015 Oct 16;10(10):e0140759. doi: 10.1371/journal.pone.0140759 (PMC4608688; doi:10.1371/journal.pone.0140759)
Supplement: S2 Text — Justification and description of the subsampling approach described in the Discussion. (PDF) [file pone.0140759.s002.pdf]

S2 Text: Detection of Diffusion Heterogeneity in Single Particle Tracking Trajectories using a Hidden Markov Model with Measurement Noise Propagation

Subsampling trajectories to reduce the effect of measurement noise

We showed (Fig. 1) that stationary beads show fast switching between two distinct diffusion coefficients, and prefer a two-state diffusion model in a model selection analysis. To address this problem, we tried subsampling the data to increase the S/N ratio, and hence minimise bias caused by the inherent two-state diffusion behaviour observed on stationary beads. This should enable differentiation between real biological switching between states with different mobilities, and artificial two diffusion behaviour.

For a trajectory $\mathbf{X}$, we subsample by taking every nth time point to obtain a trajectory of length $\lfloor \frac{N}{n} \rfloor$. Different choices of n can greatly affect the inferred parameters. Michalet provides some theoretical reasoning for the choice of subsampling rate n by determining the optimum number of points of the mean square displacement (MSD) function to include when estimating D [1]. For example, if the optimum number of MSD points is two, then the optimal D estimate comes from a linear fit to the first two MSD points (ignoring the $(0, 0)$ point). Selecting a suboptimal number of MSD points can lead to large errors.

If the optimum number of MSD points is large then single displacement estimates for D can be out by orders of magnitude [1, 2]. The D value from a single displacement analysis is equivalent to fitting a straight line from $(0, 0)$ to the first MSD point. For the trajectories in S10 Fig A (fixed latex bead) and S10 Fig B (slow moving LFA-1) the single displacement fit is not a good approximation for an MSD fit using the optimum number of points. S10 Fig C shows a fast moving LFA-1 trajectory, in this case the single displacement fit is a good approximation to the MSD fit.

Intuitively, the optimum number of MSD points should be a good approximation to the best subsampling rate. To test this the one-state and two-state diffusion model and model selection analysis (without measurement noise) was run with a subsampling rate equal to the theoretical optimum number of MSD points. S2 Table compares the preferred model from this approach to the preferred model using the one-state and two-state diffusion models with measurement noise.

References

[1] Michalet X. Mean square displacement analysis of single-particle trajectories with localization error: Brownian motion in an isotropic medium. Physical review E, Statistical, nonlinear, and soft matter physics. 2010 Oct;82(4 Pt 1):041914.

[2] Michalet X, Berglund AJ. Optimal diffusion coefficient estimation in single-particle tracking. Physical review E, Statistical, nonlinear, and soft matter physics. 2012 May;85(6 Pt 1):061916–061916.
