# Supplementary material for: Detection of Diffusion Heterogeneity in Single Particle Tracking Trajectories Using a Hidden Markov Model with Measurement Noise Propagation
Source: PLoS One. 2015 Oct 16;10(10):e0140759. doi: 10.1371/journal.pone.0140759 (PMC4608688; doi:10.1371/journal.pone.0140759)
Supplement: S1 Algorithms — Pseudocode for one-state and two-state diffusion model MCMC algorithms. (PDF) [file pone.0140759.s004.pdf]

S1 Algorithms: Detection of Diffusion Heterogeneity in Single Particle Tracking Trajectories using a Hidden Markov Model with Measurement Noise Propagation

This document contains pseudocode for one-state and two-state diffusion model MCMC algorithms.

Algorithm 1 Gibbs sampler for two-state diffusion model without measurement noise

$K \leftarrow$ number of MCMC steps
$D_{max}, a_0, b_0, a_1, b_1 \leftarrow$ choice of prior parameters (equation (7))
$D_0 \leftarrow$ random number drawn from Unif$(0, D_{max})$
$D_1 \leftarrow$ random number drawn from Unif$(0, D_{max})$
$p_{01} \leftarrow$ random number drawn from Beta(a_0, b_0)
$p_{10} \leftarrow$ random number drawn from Beta(a_1, b_1)
$\theta^{(1)} \leftarrow \{D_0, D_1, p_{01}, p_{10}\}$
$z_1 \leftarrow$ random number drawn from Bernoulli$\left(\frac{p_{10}}{p_{10}+p_{01}}\right)$
for $i = 2$ to $i = N$ **do**
  $z_i \leftarrow$ random number drawn from Bernoulli$(z_{i-1}(1-p_{10}) + (1-z_{i-1})p_{01})$
end for
$\mathbf{z}^{(1)} \leftarrow \{z_i\}_{i=1}^N$
for $k = 2$ to $k = K$ **do**
  $\eta_0 \leftarrow \sum_{z_i=0} 1$
  $\eta_1 \leftarrow \sum_{z_i=1} 1$
  if $\eta_0 = 0$ **then**
    $D_0 \leftarrow$ random number drawn from Unif$(0, D_{max})$
  else
    while $D_0 > D_{max}$ **do**
      $D_0^{-1} \leftarrow$ random number drawn from Gamma $\left(\eta_0 + 1, \sum_{i|z_i=0} \frac{\Delta X_i^2}{4\Delta t_i}\right)$
      $D_0 \leftarrow 1/D_0^{-1}$
    end while
  end if
  if $\eta_1 = 0$ **then**
    $D_1 \leftarrow$ random number drawn from Unif$(0, D_{max})$
  else
    while $D_1 > D_{max}$ **do**
      $D_1^{-1} \leftarrow$ random number drawn from Gamma $\left(\eta_1 + 1, \sum_{i|z_i=1} \frac{\Delta X_i^2}{4\Delta t_i}\right)$
      $D_1 \leftarrow 1/D_1^{-1}$
    end while
  end if
  Update $n_{10}, n_{11}, n_{01}, n_{00}$ using equation (14)
  $p_{01} \leftarrow$ random number drawn from Beta$\big(a_0 + n_{10}, b_0 + n_{11}\big)$
  $p_{10} \leftarrow$ random number drawn from Beta$\big(a_1 + n_{01}, b_1 + n_{00}\big)$
  for $i = 1$ to $i = N$ **do**
    Calculate $\pi(z_i = 1|z_{i-1}, z_{i+1}, D_0, D_1, p_{01}, p_{10}, \mathbf{X})$ by normalising equation (17), (19) or (20)
    $z_i \leftarrow$ random number drawn from Bernoulli$(\pi(z_i = 1|z_{i-1}, z_{i+1}, D_0, D_1, p_{01}, p_{10}, \mathbf{X}))$
  end for
  $\theta^{(k)} \leftarrow \{D_0, D_1, p_{01}, p_{10}\}$
  $\mathbf{z}^{(\mathbf{k})} \leftarrow \{z_i\}_{i=1}^N$
end for

Algorithm 2 Gibbs sampler for one-state diffusion model with measurement noise

$K \leftarrow$ number of MCMC steps
$D_{max}, \mu_{U_1}, \sigma_U^2 \leftarrow$ choice of prior parameters
$\sigma^2 \leftarrow$ choice of localisation accuracy
$D \leftarrow$ random number drawn from $\text{Unif}(0, D_{max})$
$D^{(1)} \leftarrow D$
$U_1 \leftarrow$ random number drawn from $N(\mu_{U_1}, \sigma_U^2)$
for $i = 2$ to $i = N + 1$ **do**
  $U_i \leftarrow$ random number drawn from $N(U_{i-1}, 2D\Delta t_{i-1})$
end for
$\mathbf{U}^{(1)} \leftarrow \{U_i\}_{i=1}^{N+1}$
for $k = 2$ to $k = K$ **do**
  $D^{-1} \leftarrow$ random number drawn from Gamma $\left(N + 1, \sum_{i=1}^{N} \frac{\Delta U_i^2}{4\Delta t_i}\right)$
  while $D > D_{max}$ **do**
    $D^{-1} \leftarrow$ random number drawn from Gamma $\left(N + 1, \sum_{i=1}^{N} \frac{\Delta U_i^2}{4\Delta t_i}\right)$
  end while
  $D^{(k)} \leftarrow D$
  for $i = 1$ to $i = N + 1$ **do**
    Calculate μ_i, τ_i from equations (31), (32) or (33)
    $U_i \leftarrow$ random number drawn from $N(\mu_i, 1/\tau_i)$
  end for
  $\mathbf{U}^{(k)} \leftarrow \{U_i^{(k)}\}_{i=1}^{N+1}$
end for

Algorithm 3 Gibbs sampler for two-state diffusion model with measurement noise

$K \leftarrow$ number of MCMC steps
$D_{max}, a_0, b_0, a_1, b_1, \mu_{U_1}, \sigma_U^2 \leftarrow$ choice of prior parameters (equation (40))
$\sigma^2 \leftarrow$ choice of localisation accuracy
$D_0 \leftarrow$ random number drawn from Unif$(0, D_{max})$
$D_1 \leftarrow$ random number drawn from Unif$(0, D_{max})$
$p_{01} \leftarrow$ random number drawn from Beta(a_0, b_0)
$p_{10} \leftarrow$ random number drawn from Beta(a_1, b_1)
$\theta^{(1)} \leftarrow \{D_0, D_1, p_{01}, p_{10}\}$
$z_1 \leftarrow$ random number drawn from Bernoulli$\left(\frac{p_{10}}{p_{10}+p_{01}}\right)$
for $i = 2$ to $i = N$ **do**
  $z_i^{(1)} \leftarrow$ random number drawn from Bernoulli$(z_{i-1}(1 - p_{10}) + (1 - z_{i-1})p_{01})$
end for
$\mathbf{z}^{(1)} \leftarrow \{z_i\}_{i=1}^N$
$U_1 \leftarrow$ random number drawn from $N(\mu_{U_1}, \sigma_U^2)$
for $i = 2$ to $i = N + 1$ **do**
  $U_i \leftarrow$ random number drawn from $N(U_{i-1}, 2D_{z_{i-1}}\Delta t_{i-1})$
end for
$\mathbf{U}^{(1)} \leftarrow \{U_i\}_{i=1}^{N+1}$
for $k = 2$ to $k = K$ **do**
  $\eta_0 \leftarrow \sum_{z_i=0} 1$
  $\eta_1 \leftarrow \sum_{z_i=1} 1$
  if $\eta_0 = 0$ **then**
    $D_0 \leftarrow$ random number drawn from Unif$(0, D_{max})$
  else
    while $D_0 > D_{max}$ **do**
      $D_0^{-1} \leftarrow$ random number drawn from Gamma $\left(\eta_0 + 1, \sum_{i|z_i=0} \frac{\Delta U_i^2}{4\Delta t_i}\right)$
      $D_0 \leftarrow 1/D_0^{-1}$
    end while
  end if
  if $\eta_1 = 0$ **then**
    $D_1 \leftarrow$ random number drawn from Unif$(0, D_{max})$
  else
    while $D_1 > D_{max}$ **do**
      $D_1^{-1} \leftarrow$ random number drawn from Gamma $\left(\eta_1 + 1, \sum_{i|z_i=1} \frac{\Delta U_i^2}{4\Delta t_i}\right)$
      $D_1 \leftarrow 1/D_1^{-1}$
    end while
  end if
  Update $n_{10}, n_{11}, n_{01}, n_{00}$ using equation (14)
  $p_{01} \leftarrow$ random number drawn from Beta$\big(a_0 + n_{10}, b_0 + n_{11}\big)$
  $p_{10} \leftarrow$ random number drawn from Beta$\big(a_1 + n_{01}, b_1 + n_{00}\big)$
  $\theta^{(k)} \leftarrow \{D_0, D_1, p_{01}, p_{10}\}$
  for $i = 1$ to $i = N$ **do**
    Calculate $\pi(z_i = 1|z_{i-1}, z_{i+1}, D_0, D_1, p_{01}, p_{10}, \mathbf{U})$ by normalising equation (43), (44) or (45)
    $z_i \leftarrow$ random number drawn from Bernoulli$(\pi(z_i = 1|z_{i-1}, z_{i+1}, D_0, D_1, p_{01}, p_{10}, \mathbf{U}))$
  end for
  $\mathbf{z}^{(\mathbf{k})} \leftarrow \{z_i\}_{i=1}^N$
  for $i = 1$ to $i = N + 1$ **do**
    Calculate μ_i, τ_i from equation (47), (48) or (49)
    $U_i \leftarrow$ random number drawn from $N(\mu_i, 1/\tau_i)$
  end for
  $\mathbf{U}^{(k)} \leftarrow \{U_i\}_{i=1}^{N+1}$
end for

Algorithm 4 Metropolis-Hastings sampler for one-state diffusion model with measurement noise incorporated as independent displacements (approximate model)

$K \leftarrow$ number of MCMC steps
$D_{max} \leftarrow$ choice of prior parameters
$\sigma^2 \leftarrow$ choice of localisation accuracy
$S_D \leftarrow$ variance of proposal distribution
$D \leftarrow$ random number drawn from $\text{Unif}(0, D_{max})$
$D^{(1)} \leftarrow D$
for $k = 2 : K$ **do**
  $D' \leftarrow$ random number drawn from $N(D, S_D)$
  $\alpha(D \to D') = \min \left\{ 1, \frac{\prod_{i=1}^{N} N\left(\Delta X_i; 0, 2(D'\Delta t_i + \sigma^2)\right)}{\prod_{i=1}^{N} N(\Delta X_i; 0, 2(D\Delta t_i + \sigma^2))} \right\} \mathbb{1}_{[0, D_{max}]}(D)$
  $u \leftarrow$ random number drawn from $\text{Unif}(0, 1)$
  if $\alpha(D \to D') > 1$ **then**
    $D \leftarrow D'$
  else if $\alpha(D \to D') > u$ **then**
    $D \leftarrow D'$
  end if
  $D^{(k)} \leftarrow D$
end for

Algorithm 5 Metropolis-within-Gibbs sampler for two-state diffusion model with measurement noise incorporated as independent displacements (approximate model)

$K \leftarrow$ number of MCMC steps
$D_{max}, a_0, b_0, a_1, b_1 \leftarrow$ choice of prior parameters
$\sigma^2 \leftarrow$ choice of localisation accuracy
$S_{D_0}, S_{D_1} \leftarrow$ variance of proposal distributions
$D_0 \leftarrow$ random number drawn from Unif$(0, D_{max})$
$D_1 \leftarrow$ random number drawn from Unif$(0, D_{max})$
$p_{01} \leftarrow$ random number drawn from Beta(a_0, b_0)
$p_{10} \leftarrow$ random number drawn from Beta(a_1, b_1)
$\theta^{(1)} \leftarrow \{D_0, D_1, p_{01}, p_{10}\}$
$z_1 \leftarrow$ random number drawn from Bernoulli$\left(\frac{p_{10}}{p_{10}+p_{01}}\right)$
for $i = 2$ to $i = N$ **do**
    $z_i \leftarrow$ random number drawn from Bernoulli$(z_{i-1}(1-p_{10}) + (1-z_{i-1})p_{01})$
end for
$\mathbf{z} \leftarrow \{z_i\}_{i=1}^N$
for $k = 2$ to $k = K$ **do**
    $D_0' \leftarrow$ random number drawn from $N(D_0, S_{D_0})$
    $\alpha(D_0 \to D_0'|\mathbf{z}, \mathbf{X}) \leftarrow \min\left\{1, \frac{\prod_{z_i=0} N(\Delta X_i; 0, 2D_0'\Delta t_i + 2\sigma^2)}{\prod_{z_i=0} N(\Delta X_i; 0, 2D_0\Delta t_i + 2\sigma^2)}\right\} \mathbb{1}_{[0,D_{max}]}(D_0)$
    $u \leftarrow$ random number drawn from Unif$(0, 1)$
    if $\alpha(D_0 \to D_0'|\mathbf{z}, \mathbf{X}) > 1$ **then**
        $D_0 \leftarrow D_0'$
    else if $\alpha(D_0 \to D_0'|\mathbf{z}, \mathbf{X}) > u$ **then**
        $D_0 \leftarrow D_0'$
    end if
    $D_1' \leftarrow$ random number drawn from $N(D_1, S_{D_1})$
    $\alpha(D_1 \to D_1'|\mathbf{z}, \mathbf{X}) \leftarrow \min\left\{1, \frac{\prod_{z_i=1} N(\Delta X_i; 0, 2D_1'\Delta t_i + 2\sigma^2)}{\prod_{z_i=1} N(\Delta X_i; 0, 2D_1\Delta t_i + 2\sigma^2)}\right\} \mathbb{1}_{[0,D_{max}]}(D_1)$
    $u \leftarrow$ random number drawn from Unif$(0, 1)$
    if $\alpha(D_1 \to D_1'|\mathbf{z}, \mathbf{X}) > 1$ **then**
        $D_1 \leftarrow D_1'$
    else if $\alpha(D_1 \to D_1'|\mathbf{z}, \mathbf{X}) > u$ **then**
        $D_1 \leftarrow D_1'$
    end if
    Update $n_{10}, n_{11}, n_{01}, n_{00}$ using equation (14)
    $p_{01} \leftarrow$ random number drawn from Beta$\big(a_0 + n_{10}, b_0 + n_{11}\big)$
    $p_{10} \leftarrow$ random number drawn from Beta$\big(a_1 + n_{01}, b_1 + n_{00}\big)$
    $\theta^{(k)} \leftarrow \{D_0, D_1, p_{01}, p_{10}\}$
    for $i = 1$ to $i = N$ **do**
        Calculate $\pi(z_i = 1|z_{i-1}, z_{i+1}, D_0, D_1, p_{01}, p_{10}, \mathbf{X})$ by normalising equation (53), (54) or (55)
        $z_i \leftarrow$ random number drawn from Bernoulli$(\pi(z_i = 1|z_{i-1}, z_{i+1}, D_0, D_1, p_{01}, p_{10}, \mathbf{X}))$
    end for
    $\mathbf{z}^{(\mathbf{k})} \leftarrow \mathbf{z}$
end for
