# Supplementary material for: Detection of Diffusion Heterogeneity in Single Particle Tracking Trajectories Using a Hidden Markov Model with Measurement Noise Propagation
Source: PLoS One. 2015 Oct 16;10(10):e0140759. doi: 10.1371/journal.pone.0140759 (PMC4608688; doi:10.1371/journal.pone.0140759)
Supplement: S1 Table — (PDF) [file pone.0140759.s005.pdf]

S1 Table. Model selection results for different Bayes factor (BF) thresholds.

**Treatment**	DMSO	Cyto D	PMA	PMA+Cal-I
**Number of trajectories**	75	36	39	46
**Two-state model preferred, BF threshold 0[1]**	18/75 (24%)	11/36 (31%)	14/39 (36%)	8/46 (17%)
**Two-state model preferred, BF threshold 0[1], fast switchers removed[2]**	13/70 (19%)	3/28 (11%)	8/33 (24%)	6/44 (14%)
**Two-state model preferred, BF threshold ±5[3]**	15/66 (23%)	7/27 (26%)	13/36 (36%)	7/42 (17%)
**Two-state model preferred, BF threshold ±5[3], fast switchers removed[2]**	12/63 (19%)	3/23 (13%)	8/31 (26%)	5/40 (13%)

1. Model selection between approximate one-state and two-state diffusion models with measurement noise, with $\log_e B_{1D,2D} < 0$ preference for the two-state model and $\log_e B_{1D,2D} > 0$ preference for the one-state model.
2. Fast switching trajectories ($\hat{p}_{01} > 0.1$ or $\hat{p}_{10} > 0.1$) also removed.
3. Model selection between approximate one-state and two-state diffusion models with measurement noise, with $\log_e B_{1D,2D} < -5$ preference for the two-state model and $\log_e B_{1D,2D} > 5$ preference for the one-state model.
