# Supplementary material for: Detection of Diffusion Heterogeneity in Single Particle Tracking Trajectories Using a Hidden Markov Model with Measurement Noise Propagation
Source: PLoS One. 2015 Oct 16;10(10):e0140759. doi: 10.1371/journal.pone.0140759 (PMC4608688; doi:10.1371/journal.pone.0140759)
Supplement: S2 Table — (PDF) [file pone.0140759.s006.pdf]

S2 Table. Influence of noise and S/N on model selection preferences between one-state and two-state diffusion models on LFA-1 data (157 trajectories).

**Models**	**No preference**	**One-state diffusion**	**Two-state diffusion**
Approximate measurement noise	19 (9.7%)	132 (67%)	45 (23%)
No measurement noise and subsampling	15 (7.7%)	130 (66%)	51 (26%)
No measurement noise	11 (5.6%)	85 (43%)	100 (51%)

Approximate measurement noise refers to model selection between one-state and two-state diffusion models with measurement noise incorporated as equations (26) and (38) (with $\sigma^2 = 41.09\text{nm}^2$). No measurement noise is model selection between one-state and two-state diffusion models without measurement noise, posterior as equations (2) and (9). No measurement noise and subsampling is model selection between one-state and two-state diffusion models without measurement noise, as equations (2) and (9), but on trajectories subsampled at a rate equal to the optimum number of MSD points for estimating D, see S2 Text. For all models the MCMC runs were 20000 steps with a 10000 step burn-in. See Methods for priors and initial conditions.
