# Supplementary material for: Detection of Diffusion Heterogeneity in Single Particle Tracking Trajectories Using a Hidden Markov Model with Measurement Noise Propagation
Source: PLoS One. 2015 Oct 16;10(10):e0140759. doi: 10.1371/journal.pone.0140759 (PMC4608688; doi:10.1371/journal.pone.0140759)
Supplement: S3 Table — (PDF) [file pone.0140759.s007.pdf]

S3 Table. Comparison of model selection for approximate measurement noise models and subsampled trajectories.

		**Approximate measurement noise**		
		No preference	One-state diffusion	Two-state diffusion
**No measurement noise and subsampling**	No preference	5	6	4
	One-state diffusion	13	108	9
	Two-state diffusion	1	18	32

For approximate measurement noise the model selection was between one-state and two-state diffusion models with measurement noise, described in Methods. For no measurement noise and subsampling the model selection was between one-state and two-state diffusion models without measurement noise, as described in Methods but with trajectories subsampled at a rate equal to optimum number of MSD points to include when estimating D, see S2 Text. For all models the MCMC runs were 20000 steps with a 10000 step burn-in. See Methods for priors and initial conditions.
